# Deletion of the Pluripotency-Associated *Tex19.1* Gene Causes Activation of Endogenous Retroviruses and Defective Spermatogenesis in Mice

**DOI:** 10.1371/journal.pgen.1000199

**Published:** 2008-09-19

**Authors:** Rupert Öllinger, Andrew J. Childs, Hannah M. Burgess, Robert M. Speed, Pia R. Lundegaard, Nicola Reynolds, Nicola K. Gray, Howard J. Cooke, Ian R. Adams

**Affiliations:** 1 MRC Human Genetics Unit, Western General Hospital, Edinburgh, United Kingdom; 2 School of Clinical Sciences and Community Health, University of Edinburgh, Edinburgh, United Kingdom; 3 MRC Human Reproductive Sciences Unit, Centre for Reproductive Biology, The Queen's Medical Research Institute, Edinburgh, United Kingdom; 4 Institute for Genetics and Molecular Medicine, Western General Hospital, Edinburgh, United Kingdom; 5 Edinburgh Cancer Research Centre, School of Molecular and Clinical Medicine, University of Edinburgh, Western General Hospital, Edinburgh, United Kingdom; Massachusetts General Hospital, Howard Hughes Medical Institute, United States of America

## Abstract

As genetic information is transmitted through successive generations, it passes between pluripotent cells in the early embryo and germ cells in the developing foetus and adult animal. *Tex19.1* encodes a protein of unknown function, whose expression is restricted to germ cells and pluripotent cells. During male spermatogenesis, *Tex19.1* expression is highest in mitotic spermatogonia and diminishes as these cells differentiate and progress through meiosis. In pluripotent stem cells, *Tex19.1* expression is also downregulated upon differentiation. However, it is not clear whether *Tex19.1* has an essential function in germ cells or pluripotent stem cells, or what that function might be. To analyse the potential role of *Tex19.1* in pluripotency or germ cell function we have generated *Tex19.1^−/−^* knockout mice and analysed the *Tex19.1^−/−^* mutant phenotype. Adult *Tex19.1^−/−^* knockout males exhibit impaired spermatogenesis. Immunostaining and histological analysis revealed defects in meiotic chromosome synapsis, the persistence of DNA double-strand breaks during meiosis, and a loss of post-meiotic germ cells in the testis. Furthermore, expression of a class of endogenous retroviruses is upregulated during meiosis in the *Tex19.1^−/−^* testes. Increased transposition of endogenous retroviruses in the germline of *Tex19.1^−/−^* mutant mice, and the concomitant increase in DNA damage, may be sufficient to disrupt the normal processes of recombination and chromosome synapsis during meiosis and cause defects in spermatogenesis. Our results suggest that *Tex19.1* is part of a specialised mechanism that operates in the germline to repress transposable genetic elements and maintain genomic stability through successive generations.

## Author Summary

The germ cells—eggs in females and sperm in males—are responsible for passing genetic information from one generation to the next. As any genetic changes that arise in the germ cells can be transmitted to the next generation, germ cells are a prime target for the activity of mobile genetic elements. Mobile genetic elements make up around 40% of a mammalian genome, and many of these elements are derived from retroviruses that have infected germ cells, or early embryonic precursors to germ cells, and have integrated into the genome. Here, we characterise the function of *Tex19.1*, a gene whose expression is restricted to germ cells and the pluripotent cells that are early embryonic precursors to germ cells. We show that when *Tex19.1* is deleted from mice, germ cells have problems progressing through meiosis, and sperm production is impaired. Furthermore, we show that, in the absence of *Tex19.1*, endogenous retroviruses are activated in male germ cells attempting to go through meiosis. Our results suggest that *Tex19.1* is part of a specialised mechanism that guards against mutagenic endogenous retrovirus activity in germ cells and pluripotent cells and thus helps to maintain the integrity and stability of the genome through successive generations.

## Introduction

The germ cells of sexually reproducing organisms have a unique role in generating genetic diversity and transmitting genetic information from one generation to the next. Establishment of the germline in mammals involves the induction of germ cells from pluripotent epiblast cells through the action of extra-embryonic ectoderm-derived bone morphogenetic proteins and occurs comparatively late in development, commencing around day 6.25 days post coitum (dpc) in mice [1]–[4]. At around 12.5 dpc -13.5 dpc the sexually dimorphic germ cells become committed to develop along either a male or a female pathway and start to initiate sex-specific differentiation [5]. Although there are numerous differences in the differentiation of the germline and in the timing and regulation of meiosis between the sexes, the fundamental events of meiosis that increase genetic diversity and reduce ploidy of the gametes are common to both.

The main group of genes that have been shown to be required for mammalian meiosis are those involved in the recombination and synapsis of homologous chromosomes. Mice carrying loss-of-function mutations in these genes, such as *Atm*, *Dmc1*, *γH2AX, Mlh1*, *Msh5*, *Rec8*, *Rad51*, *Smc1β*, *Spo11*, *Sycp1*, *Sycp2*, *Sycp3, Syce2* and *Tex14*, typically exhibit defects in chromosome synapsis in both sexes, although male and female germ cells can exhibit different responses to these defects [6]–[8].

A second group of genes that are required for progression through meiosis are those involved in repression of transposable genetic elements. Retrotransposons, for example long interspersed repeats (LINEs), short interspersed repeats (SINEs), and endogenous retroviruses such as intracisternal A-particles (IAPs) are the major class of transposable genetic elements in mammals and comprise around 37.5% of the mouse genome [9]. To allow new transposition events to propagate through subsequent generations, retrotransposons have evolved to be active in the germline. Accordingly, germ cells appear to have evolved mechanisms to reduce the mutational load of retrotransposon activity. Mutations in genes involved in mediating DNA methylation-dependent transcriptional repression of retrotransposons cause increased expression of retrotransposons and defects in chromosome synapsis during meiosis in either male or female germ cells [10],[11]. For example Dnmt3L is a catalytically inactive member of the DNA methyltransferase family that is expressed in foetal germ cells but is absent by 6 days post partum (dpp) [11]. Male mice null for this gene do not methylate dispersed repeat DNA during foetal germ cell development, and express LINEs and a class of endogenous retroviruses known as intracisternal A-particles (IAPs) in the germline [11]. *Dnmt3L* mutant male mice also exhibit meiotic abnormalities that result in a loss of post-meiotic germ cells in the testis [11]. The *Dnmt3L* mutant phenotype suggests that epigenetic changes that occur in foetal germ cells can cause meiotic defects later in germ cell development. A second example is provided by the murine piwi-related genes, which encode germline-specific proteins that are associated with a class of small germline-specific piwi-interacting RNAs (piRNAs) and are also required to repress retrotransposons during spermatogenesis [12]–[14]. *Mili* and *Miwi2* both appear to be involved in de novo methylation of LINE and IAP elements during germ cell development in male embryos, and both mutants exhibit reduced DNA methylation and increased expression of LINE and IAP element in the testis, and defects in chromosome synapsis during meiosis in male germ cells [12]–[14]. The mechanism by which increased expression of retrotransposons results in the defects in chromosome synapsis seen in these mutant mice is unknown, but the phenotype of these mutant mice suggests that repression of transposable genetic elements is required to allow germ cells to progress through meiosis.

A set of testis expressed (*Tex*) genes has been identified in a subtractive hybridisation screen for genes expressed in spermatogonia but not somatic tissue [15]. One of these genes, *Tex19.1* (AAH53492.1), was found in a screen for potential RNA-targets of the germline-specific RNA binding protein Dazl by immunoprecipitation and microarray analysis [16]. Further unpublished work in this laboratory and work recently published by Kuntz et al. [17] confirmed an earlier report that *Tex19.1* is a “pluripotent cell expressed gene” [18]. Humans and primates possess a single *Tex19* gene in their genome, but in rodents a recent duplication has produced a two-gene family arranged as divergently transcribed genes separated by 29kb of DNA [17]. While expression of murine *Tex19.1* is restricted to pluripotent stem cells and developing germ cells, *Tex19.2* is expressed in the testis somatic tissues and does not appear to be restricted to germ cells or pluripotent stem cells in mice [17].

The expression pattern of *Tex19.1* suggests that the protein could have an important role in pluripotency or germ cell function. Since the sequence of Tex19.1 gives no clue to the biochemical function of this protein we decided to take a genetic approach to determining the function of this gene in the germline. In this paper we report that targeted deletion of *Tex19.1* in mice results in upregulation of endogenous retrovirus expression in testicular germ cells, perturbed chromosome synapsis during meiosis, and impaired spermatogenesis.

## Materials and Methods

### Generation of *Tex19.1* Knockout Mice

*Tex19.1* knockout mice were generated by replacing the *Tex19.1* open reading frame with a neomycin selection cassette by homologous recombination in E14 embryonic stem cells [19]. Homologous regions were cloned by PCR from E14 embryonic stem cell genomic DNA using primers listed in Supplementary Table S1. The *Tex19.1* targeting vector was linearised, electroporated into E14 embryonic stem cells, and neomycin-resistant clones screened for the desired integration event by PCR and Southern blot. *Tex19.1^+/−^* ES cells were used to generate *Tex19.1^−/−^* knockout mice by blastocyst injection and breeding as described [19]. Mice were genotyped by multiplex PCR using primers listed in Supplementary Table S1. Phenotypic analysis was performed on mice with a 129/Ola x CD1 mixed genetic background. Generation and analysis of *Tex19.1^−/−^* knockout mice was performed under a UK Home Office project licence with approval from an institutional ethics committee.

### Antibody Production

Anti-Tex19.1 antibodies were raised in rabbits using the synthetic peptide ^78^ESEQEPGPEQDAWRG^92^ (Eurogentec). This peptide was designed to be specific to Tex19.1 and is not present in the Tex19.2 protein sequence. Antibodies were affinity purified from sera using the immunising peptide immobilised on a Sulfolink column (Pierce) according to manufacturer's instructions.

### Immunohistochemistry

Testes were recovered from mice, fixed at 4°C overnight in 4% paraformaldehyde in phosphate buffered saline (PBS) and embedded in paraffin wax. 6 µm-thick sections were dewaxed in xylene, rehydrated, and antigen retrieval performed by boiling slides for 15 minutes in 0.01 M sodium citrate, pH 6.0. Sections were blocked, incubated with rabbit anti-Tex19.1 primary antibody at 1∶50, and bound antibody detected using the DAKOvision ABC diaminobenzidine (DAB) kit as described by the manufacturer (DakoCytomation). For peptide competition, anti-Tex19.1 antibodies were pre-incubated with 5 nM immunising peptide. For immunostaining of cultured cells, cells were fixed for 30 minutes at room temperature with 3.7% formaldehyde in PBS, then blocked with PBS containing 5% serum and 0.01% Tween-20. Cells were incubated with rabbit anti-Tex19.1 primary antibody at 1∶100, then fluorescently labelled secondary antibodies at 1 µg/mL (Invitrogen). DNA was counterstained with 2 µg/mL DAPI.

### Nuclear/Cytoplasmic Fractionation

E14 embryonic stem cells or 13 dpp postnatal testes were lysed in cytoplasmic lysis buffer (10 mM Hepes pH 7.6, 3 mM MgCl_2_, 40 mM KCl, 50 mM β-glycerophosphate, 5% glycerol, 0.5% Igepal CA-630, 2 mM NaF, 1 mM Na_3_VO_4_, 2 mM DTT and protease inhibitors) for 5 minutes on ice and the whole cell lysate centrifuged for 5 minutes at 1000g at 4°C. The nuclear pellet was resuspended in Laemmli buffer, boiled for 5 minutes and sonicated to disrupt genomic DNA. The cytosolic supernatant was mixed with Laemmli buffer and boiled for 5 minutes. Equivalent proportions of each fraction were separated by SDS-PAGE then Western blotted.

### Western Blot

Testis was homogenised in Laemmli buffer, boiled for 5 min and sonicated to disrupt genomic DNA. Western blotting was performed using standard procedures [20]. Tex19.1 was detected with rabbit anti-Tex19.1 polyclonal antibodies used at a 1∶200 dilution, mouse anti-Gapdh antibodies (Abcam) were used at 1∶1000, mouse anti-HP1α antibodies (Chemicon) at 1∶2500, and rabbit anti-histone H3 antibodies (Abcam) at 1∶20000. Peroxidase-conjugated secondary antibodies and enhanced chemiluminescence were used to detect primary antibodies.

### Southern Blotting

Non-radioactive Southern blots were performed using a digoxigenin-labeled DNA probe generated using primers listed in Supplementary Table S1, and alkaline phosphatase-conjugated anti-digoxigenin antibodies, essentially as described by the manufacturer (Roche).

### RT-PCR and Quantitative PCR

Testis RNA was isolated with Trizol (Invitrogen) according to the manufacturer's protocol and reverse transcription performed with Superscript III (Invitrogen) on 1 µg RNA per reaction using oligo dT primer. Primers for RT-PCR are listed in Supplementary Table S1.

For quantitative PCR (qPCR), random-primed cDNA was generated from total RNA using Superscript III (Invitrogen). qPCR was performed using SYBR Green PCR System (Applied Biosystems) and a PTC-200 thermal cycler equipped with a Chromo4 continuous fluorescence detector and Opticon Monitor software (MJ Research). Primers for qPCR are listed in Supplementary Table S1. Five technical replicates were performed for each biological sample, and the relative changes in gene expression determined using the ΔΔ^−2Ct^ method as described [21]. As *Tex19.1* is expressed in the germ cells in the testis the Sertoli cell marker *Sdmg1* [22] was used to normalise cDNAs prepared from different animals to reduce the probability that the cDNAs were being normalised to a transcript whose level could be influenced by loss of *Tex19.1.*

### Testis Weight and Sperm Count

Both testes from each adult animal (6–36 weeks old) were weighed, and the mean testis weight was used for statistical comparison. For sperm count one epididymis from each animal was homogenised in 1 mL 1% sodium citrate and incubated for 5 minutes at room temperature to allow the debris to settle. Sperm in the supernatant was then counted with a hemocytometer.

### Histology

Testes were fixed for 4–6 hours in Bouin's solution (Sigma-Aldrich) at room temperature, then embedded in wax. For histological analysis 6 µm sections were dewaxed with xylene, rehydrated, then stained with hematoxylin and eosin.

### Immunostaining of Meiotic Chromosome Spreads

Immunostaining of chromosome spreads from meiotic spermatocytes was performed essentially as described [23]. Briefly, testes were homogenized in PBS and 0.1 mL of cells were incubated in 0.5 mL 5% sucrose on a microscope slide for 1 hour. Cells were lysed with 0.1 mL 0.05% Triton-X-100 for 10 minutes, and fixed with 0.8 mL of fixing solution (2% paraformaldehyde, 0.02% SDS in PBS) for 1 hour. The slides were then washed, blocked with 5% serum, 0.1% Tween in PBS and incubated with primary antibodies for 1 hour. Mouse anti-Sycp3 antibodies (Abcam) were used at a 1∶2000 dilution, rabbit anti-Sycp1 antibodies (Abcam) at 1∶250, rabbit anti-γH2AX antibodies (Upstate Biotechnology) at 1∶200, and mouse anti-Rad51 antibodies (Upstate Biotechnology) at 1∶125. Fluorescently labelled secondary antibodies were used at 1 µg/mL (Invitrogen), and DNA was stained with 2 µg/mL DAPI.

### Metaphase I Analysis of Spermatocytes

Chromosome spreads for metaphase I analysis were prepared as described in [24]. Briefly, testes were incubated for 20 minutes in 1% sodium citrate, minced with scissors, and the cells harvested by centrifugation. Cells were then washed and resuspended in fixing solution (3∶1 methanol∶glacial acetic acid), dropped onto slides, and the resulting chromosome spreads were stained with Giemsa solution. 100 metaphase I spreads were scored per animal, and two animals scored for each genotype.

### In Situ Hybridisation and Northern Blotting

A 460 bp fragment of the MMERVK10C endogenous retrovirus was amplified by RT-PCR from *Tex19.1^−/−^* mutant testes using primers listed in Supplementary Table S1 and cloned into pBluescript II SK+ (Stratagene). Sense and anti-sense digoxigenin-labelled riboprobes were generated using T3 and T7 RNA polymerase according to the supplier's instructions (Roche). In situ hybridisation on 6 µm wax sections of Bouin's-fixed testis tissue was performed essentially as described [25] using 100 ng/mL digoxigenin-labelled probe and a hybridisation temperature of 50°C. Bound probe was detected with alkaline phosphatase-conjugated anti-digoxigenin antibodies (Roche) and BCIP/NBT precipitating stain (Vector Labs), then sections counterstained with nuclear fast red according to manufacturer's instructions. The antisense MMERVK10C digoxigenin-labelled riboprobe was also used for non-radioactive Northern blotting of 1 µg testis RNA as described [22].

## Results

### Expression of Tex19.1 Protein during Spermatogenesis

**Figure 1:**
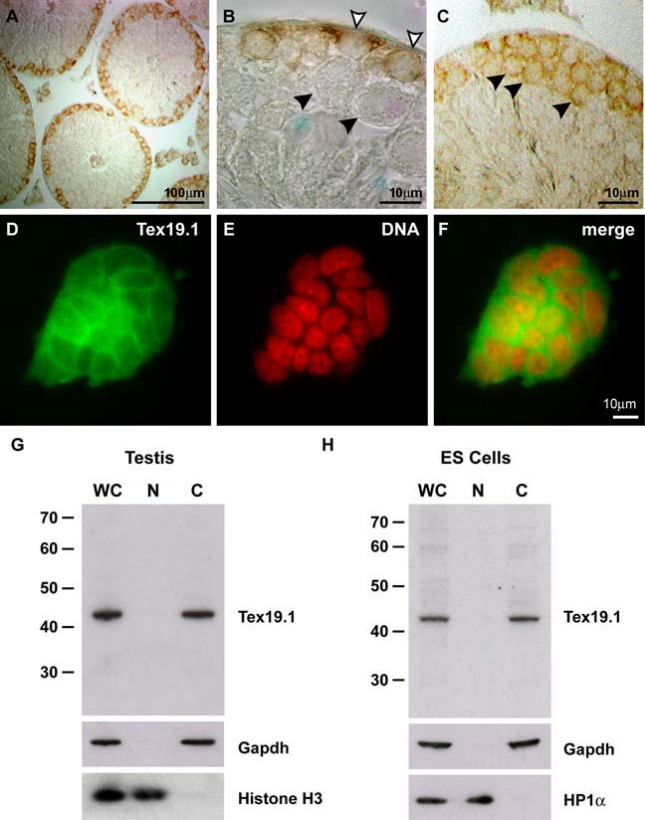
Tex19.1 is a cytoplasmic protein expressed in spermatogonia and spermatocytes in the adult testis. (A–C) Immunohistochemistry for Tex19.1 in adult testis. Anti-Tex19.1 staining (brown precipitate) can be seen in the cytoplasm of spermatogonia (open arrowheads in B) and early spermatocytes (filled arrowheads in C) in the seminiferous tubules but not in later stage pachytene spermatocytes (filled arrowheads in B). (D–F) Immunofluorescence for Tex19.1 in embryonic stem cells. Anti-Tex19.1 staining (green) can be seen in the cytoplasm of embryonic stem cells. DNA is counterstained in red. (G) Western blot for Tex19.1 in whole cell (WC), nuclear (N) and cytoplasmic (C) fractions from 13 dpp prepubertal testis. Subcellular fractionation was monitored using histone H3 and Gapdh as nuclear and cytoplasmic markers respectively. Tex19.1 is detected predominantly in the cytoplasm. (H) Western blot for Tex19.1 in whole cell, nuclear and cytoplasmic fractions from embryonic stem cells. Tex19.1 is predominantly cytoplasmic, and the purity of the fractions is shown by the cytoplasmic marker Gapdh and the nuclear marker HP1α.

Spermatogenesis in the adult testis involves the differentiation of a small pool of spermatogonial stem cells into large numbers of mature sperm. Within the testis spermatogenesis takes place in the seminiferous tubules where the mitotic spermatogonia reside at the outermost edge of the tubule, and progressive stages of differentiation are found as layers of meiotic spermatocytes then haploid spermatids located more and more centrally towards the lumen of the tubule [26]. Published RT-PCR expression data for *Tex19.1* in purified spermatogenic cell populations suggests that *Tex19.1* expression is highest in mitotic spermatogonia, decreases as the spermatocytes progress through meiosis, and is present at low levels in round spermatids [27]. To establish the expression pattern of Tex19.1 protein in male testis during spermatogenesis we raised anti-peptide antibodies to Tex19.1. Immunohistochemistry on mouse testis shows strong cytoplasmic expression of Tex19.1 in spermatogonia that is downregulated as these cells differentiate and progress through meiosis (Figure 1A–C). We were able to detect cytoplasmic Tex19.1 protein in some meiotic spermatocytes (Figure 1C), but not in others (Figure 1B) suggesting that Tex19.1 protein expression is switched off as the germ cells proceed through meiosis. The expression of Tex19.1 protein in spermatogonia and spermatocytes is consistent with that observed for *Tex19.1* mRNA by in situ hybridisation (Figure S1A–C).

Our finding that Tex19.1 is present in the cytoplasm of spermatogonia and early spermatocytes in the adult testis is not consistent with the published nuclear localisation of Tex19.1 protein in embryonic stem cells [17]. We confirmed that our anti-Tex19.1 antibody detects Tex19.1 and not a cross-reacting antigen by blocking the anti-Tex19.1 immunohistochemistry signal by competition with the immunising peptide, and by immunohistochemistry on *Tex19.1^−/−^* knockout testes (Figure S1D–F). We were also able to detect a predominantly cytoplasmic subcellular localisation of Tex19.1 by immunostaining germ cells isolated from 14.5 dpc embryonic testes (Figure S1G). Again the cytoplasmic anti-Tex19.1 immunostaining could be competed with the immunising peptide, and was absent in germ cells from *Tex19.1^−/−^* knockout embryos (Figure S1H, I). These data suggest that the anti-Tex19.1 antibody used in this present study specifically recognises endogenous Tex19.1 in germ cells, and that at least some Tex19.1 is present in the cytoplasm of spermatogonia and early spermatocytes in adult mouse testes.

To investigate whether the discrepancy between the cytoplasmic localisation of Tex19.1 in germ cells presented in this study and the nuclear localisation of Tex19.1 in embryonic stem cells described previously [17] is caused by the difference between the cell types studied we performed immunostaining for Tex19.1 on embryonic stem cells. In contrast to the previous report [17], we found that Tex19.1 is predominantly cytoplasmic in embryonic stem cells (Figure 1D–F).

**Figure 2:**
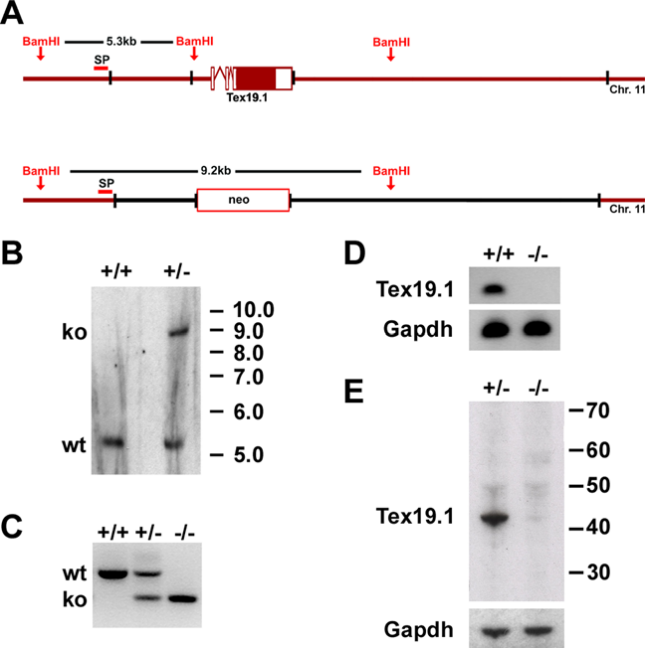
Generation of *Tex19.1^−/−^* knockout mice. (A) Schematic view of the *Tex19.1* knockout strategy. The *Tex19.1* gene in the wild-type (wt) allele is replaced by a neomycin box in the knockout (ko) allele; black borders delineate the short and long arms upstream and downstream of the gene. Location of probe for Southern blot (SP), *Bam*HI sites and lengths of restriction fragments are indicated for both alleles. (B) Southern blot of wild type E14 embryonic stem cell genomic DNA (+/+) and a successfully targeted clone (+/−). (C) Genotyping of a wild type, a heterozygous and a homozygous animal from the *Tex19.1* knockout line by multiplex PCR. (D) RT-PCR from testes of wild type and knockout animals. *Tex19.1* transcript can be detected in wild type but not in knockout testes. *Gapdh* transcript can be seen in both. (E) Western blot on protein extracts from the testes of heterozygous and knockout animals: Tex19.1 is detected in heterozygous but not in knockout testes. Gapdh is shown as a loading control.

To exclude the possibility that our anti-Tex19.1 antibody is unable to detect a nuclear population of Tex19.1 due to loss or masking of the epitope during the immunohistochemical procedures we biochemically fractionated 13 dpp prepubertal testes and embryonic stem cells into nuclear and cytoplasmic fractions. On Western blots the 42 kDa Tex19.1 band was barely detectable in the nuclear fraction, but was easily detectable in equivalent loadings of the whole cell lysate and cytoplasmic fractions (Figure 1G,H). The observed size of the anti-Tex19.1 band in the Western blots (42 kDa) correlates well with the predicted molecular weight of Tex19.1 (40.4 kDa). This 42 kDa band appears to be endogenous Tex19.1 as it is not present in testes from *Tex19.1^−/−^* knockout animals (Figure 2E). The biochemical fractionation of embryonic stem cells and testes therefore confirms the predominantly cytoplasmic subcellular localisation of Tex19.1 that we have observed by immunohistochemistry and immunostaining. Taken together the data presented here strongly suggests that Tex19.1 is a predominantly cytoplasmic protein in embryonic stem cells and germ cells.

Germ cells in many species possess specialised cytoplasmic structures termed nuage that are implicated in RNA metabolism. Although Tex19.1 appears to be a predominantly cytoplasmic germ cell protein, the subcellular localisation and cell-type distribution of Tex19.1 appears to be distinct from the nuage component Tdrd1 [28] (Figure S2). Thus Tex19.1 does not appear to be a novel component of nuage. As the subcellular localisation of Tex19.1 does not provide any major insight into what the cellular function of this protein might be, and the Tex19.1 protein sequence does not contain any functional domains to illuminate the potential biochemical function of this protein, we decided to take a genetic approach to analyse the function of *Tex19.1* in the germline.

### Generation of *Tex19.1^−/−^* Knockout Mice

In order to investigate the function of *Tex19.1* in germ cell development we generated *Tex19.1^−/−^* knockout mice. The *Tex19.1* open reading frame was replaced with a neomycin-resistance cassette by homologous recombination in embryonic stem cells (Figure 2A), and the targeted deletion confirmed by Southern blotting (Figure 2B). The heterozygous *Tex19.1^+/−^* embryonic stem cells were used to generate chimaeric mice by blastocyst injection, and the *Tex19.1^−^* mutant allele bred to homozygosity (Figure 2C). *Tex19.1^−/−^* homozygous pups were born from heterozygous crosses at a sub-Mendelian frequency (72 wild-type, 131 heterozygous, 40 homozygous pups born from heterozygous matings, significant deviation from expected Mendelian 1∶2∶1 ratio, χ^2^-test p<0.01). The low rate of recovery of *Tex19.1^−/−^* homozygous animals at birth indicates that some *Tex19.1^−/−^* homozygous embryos are lost during embryonic development.

To confirm that the *Tex19.1^−^* mutant allele removes *Tex19.1* mRNA and protein we performed RT-PCR on *Tex19.1^−/−^* testis cDNA with *Tex19.1*-specific primers (Figure 2D) and Western blotting on *Tex19.1^−/−^* testis protein extract with anti-Tex19.1 antibodies (Figure 2E). Both methods show that *Tex19.1* is not expressed in the testes of *Tex19.1^−/−^* homozygous mice. We conclude that the *Tex19.1^−^* allele that we have produced is a null allele and that *Tex19.1* function is ablated in the *Tex19.1^−/−^* homozygous mice.

**Figure 3:**
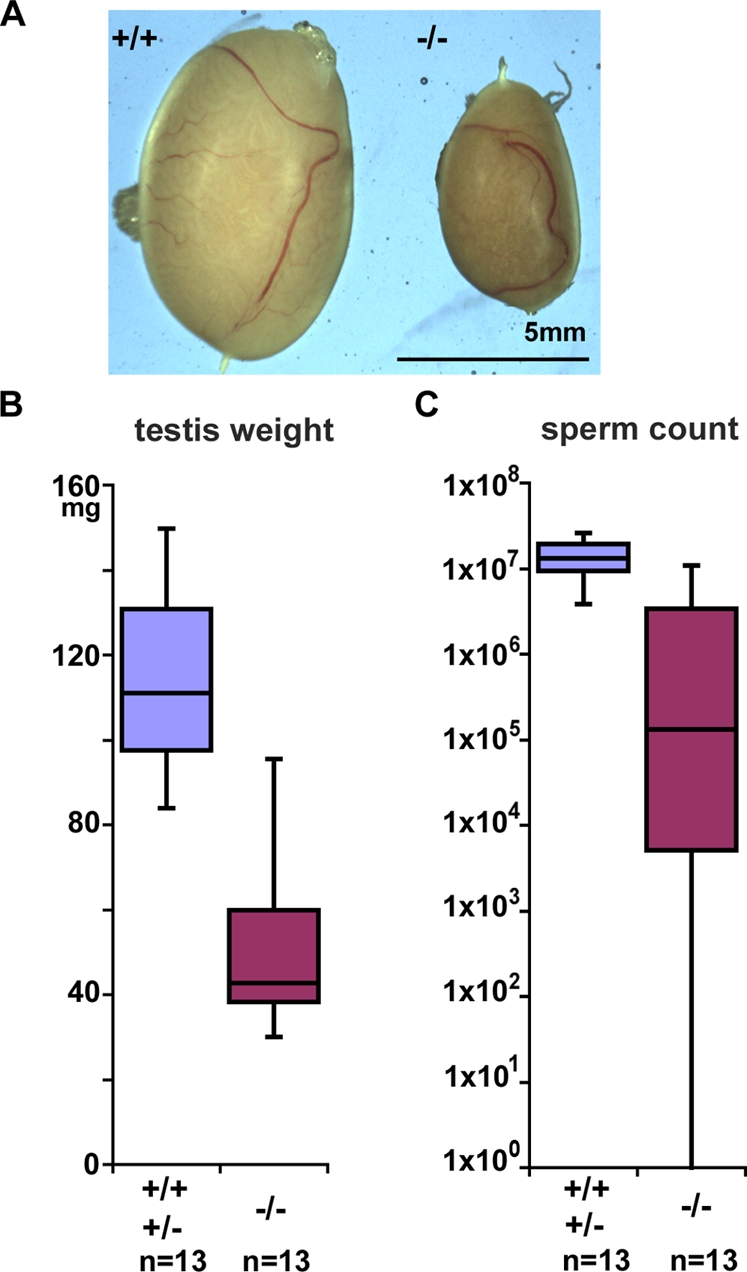
*Tex19.1^−/−^* knockout mice have defects in spermatogenesis. (A) Testes from *Tex19.1^−/−^* knockout mice are smaller in size than testes from *Tex19.1^+/+^* wild-type littermates (comparison of two 36-week old testes). (B) Box plot showing that median testis weights of adult animals are reduced in *Tex19.1^−/−^* knockout mice (Mann Whitney U-test, p<0.01). The genotypes and number of animals analysed are indicated. (C) Box plot showing that median epididymal sperm count is reduced in *Tex19.1^−/−^* knockout mice (Mann Whitney U-test, p<0.01).

The surviving *Tex19.1^−/−^* knockout mice are apparently healthy, with overtly normal morphology and behaviour. However both male and female *Tex19.1^−/−^* knockout mice have reduced fertility. *Tex19.1^−/−^* knockout females have a mean litter size of 5.0±2.2 SD, n = 9 compared to a litter size of 10.6±2.9, n = 23 for *Tex19.1^+/−^* heterozygous females (Student's t-test p<0.01). The reduced fertility in *Tex19.1^−/−^* homozygous females is consistent with expression of *Tex19.1* in embryonic ovaries [17], and a detailed analysis of the cause of the subfertility in the *Tex19.1^−/−^* knockout female mice will be published elsewhere. Similarly, *Tex19.1^−/−^* knockout male mice are also severely subfertile when test-mated with wild-type female mice. Although one out of the eleven *Tex19.1^−/−^* knockout males tested for fertility was able to sire offspring, the remaining *Tex19.1^−/−^* knockout males were infertile. The sterile *Tex19.1^−/−^* knockout male mice were apparently able to mate with the wild-type females to produce a copulation plug, but these females did not give birth to any pups. *Tex19.1^−/−^* knockout male mice have smaller testes (Figure 3A, B), with the median testis weights of adult animals reduced from 111 mg in *Tex19.1^+/+^* wild type and *Tex19.1^+/−^* heterozygous mice to 42.5 mg in *Tex19.1^−/−^* knockout littermates (Mann Whitney U-test, p<0.01). Furthermore, the median epididymal sperm count is reduced to 1.3×10^5^ in *Tex19.1^−/−^* knockout mice from 1.3×10^7^ in wild type and heterozygous littermates (Mann Whitney U-test, p<0.01, Figure 3C) suggesting that spermatogenesis is defective in the *Tex19.1^−/−^* knockout testes. We have not been able to detect any difference in testis weight or sperm count between *Tex19.1^+/+^* wild-type and *Tex19.1^+/−^* heterozygous animals.

The extent of the spermatogenesis defect in the *Tex19.1^−/−^* knockout males varied between individual animals. When sperm was counted, a strong reduction was observed for most of the animals, but for the single fertile animal the sperm count was close to normal levels (Figure 3C). A similar variation in testis weight was also evident amongst *Tex19.1^−/−^* knockout animals (Figure 3B). This phenotypic variation was not influenced by the age of the mice at the time of analysis. Age-matched adult mice were analysed at 6 weeks, 3 months, 6 months and 9 months during the course of this study, and there appeared to be no correlation between the severity of the phenotype and the age at which the adult mice were examined. Rather phenotypic variation was observed in adult mice at all ages (R.O. and I.R.A., data not shown). The outbred component of the genetic background of these mice may contribute to this variation.

### *Tex19.1^−/−^* Knockout Spermatocytes Exhibit Defects in Progression through Meiosis

**Figure 4:**
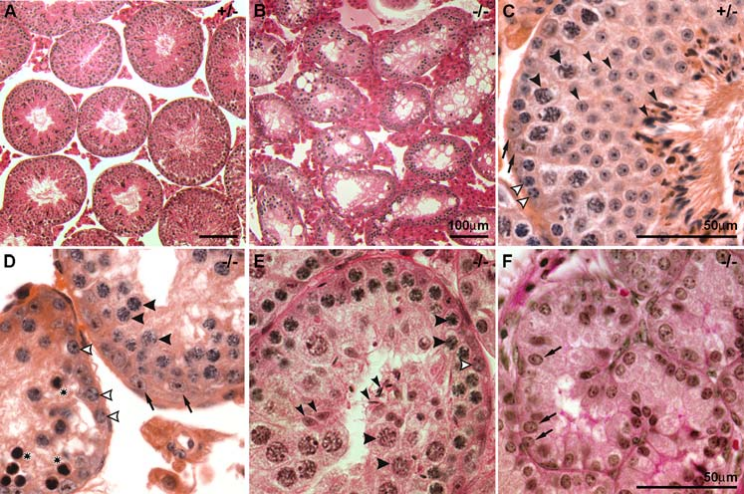
*Tex19.1^−/−^* null testes contain reduced numbers of post-meiotic germ cells. Testis histology in *Tex19.1^+/−^* heterozygous and *Tex19.1^−/−^* knockout animals. (A, C) In *Tex19.1^+/−^* heterozygotes, the testis histology is normal and the seminiferous tubules contain Sertoli cells (arrows), spermatogonia (white arrowheads), spermatocytes (broad arrowheads) and spermatids (narrow arrowheads). (B, D) *Tex19.1^−/−^* knockout animals with a severe phenotype feature a reduced tubule diameter with a substantial reduction of cell numbers. Sertoli cells (arrows) spermatogonia (open arrowheads) and meiotic cells (broad arrowheads) are present, but few spermatids can be found. Additionally, there are cells with pyknotic appearance (asterisks). (E) In *Tex19.1^−/−^* knockout animals with a less severe phenotype, some spermatids (narrow arrowheads) can be detected in reduced numbers. (F) In two of thirty *Tex19.1^−/−^* knockout animals analysed one of the recovered testes was agametic and only contained Sertoli cells (arrows). A–E are from 3 month old adult animals, F is from a 31 dpp prepubertal animal.

We investigated the spermatogenesis defect in *Tex19.1^−/^* ^−^ knockout mice further by examining the testis histology in these animals. We did not detect any overt differences in testis histology between *Tex19.1^+/+^* wild-type and *Tex19.1^+/−^* heterozygous animals. However, *Tex19.1^−/−^* knockout testes have considerably narrower seminiferous tubules than their wild-type or heterozygous littermates due to a reduction in the number of post-meiotic germ cells (Figure 4A, B). This phenotype was also subject to some heterogeneity. In animals with a more severe phenotype all postmeiotic cell-types were missing and the most advanced meiotic cells were in pachytene stage (Figure 4C, D). In animals with a less severe phenotype a proportion of cells were able to complete meiosis and haploid cells could be detected, although often in comparatively low numbers (Figure 4C, E). Like the testis weight and sperm count phenotypes described in the previous section, there appeared to be no correlation between the severity of the testis histology phenotype and the age at which the adult mice were analysed (from 6 weeks to 9 months).

To test whether the reduction in the number of post-meiotic germ cells in *Tex19.1^−/−^* knockout testes arises from a decrease in the spermatogonial mitotic divisions or apoptosis of differentiating germ cells, we counted the number of B-type spermatogonia, early meiotic cells and apoptotic cells in testis sections from *Tex19.1^+/−^* heterozygous and *Tex19.1^−/−^* knockout animals. B-spermatogonia and early meiotic cells were identified by their location and histological appearance in the seminiferous tubules [26], and apoptotic cells were identified using the TUNEL assay to label fragmented chromatin. Whereas the number of B-type spermatogonia and early meiotic cells did not differ between *Tex19.1^+/−^* heterozygous and *Tex19.1^−/−^* knockout testes (R.O., data not shown), TUNEL staining showed an increase in the number of dying cells in adult *Tex19.1^−/−^* knockout testis (Figure S3A, B, N). In more severe *Tex19.1^−/−^* knockout seminiferous tubules, TUNEL-positive cells were found within or next to layers of meiotic germ cells (Figure S3C), but even at high magnification the nuclear morphology of these TUNEL-positive cells was not distinct enough to allow their developmental stage to be unambiguously identified (Figure S3H–J). In less severe *Tex19.1^−/−^* knockout seminiferous tubules, TUNEL-positive cells could also be found between the layers of meiotic germ cells and post-meiotic round spermatids (Figure S3D). At higher magnification, some of these TUNEL-positive cells could be identified as metaphase I spermatocytes (Figure S3E–G).

In order to further define the point during spermatogenesis when the *Tex19.1^−/−^* knockout cells are dying we examined the synchronous first wave of spermatogenesis that occurs in prepubertal mice. The first wave of spermatogenic germ cells initiates meiosis at around 10 dpp in the prepubertal testis, and progresses through the pachytene stage of meiosis from around 14 to 20 dpp to produce the first post-meiotic round spermatids around 21 dpp, and mature sperm at around 31 dpp [29],[30]. Analysis of apoptosis (Figure S3N) and testis histology (Figure S4) at various stages of prepubertal testis development revealed no overt differences in testis histology and no statistically significant increase in apoptosis at 16 dpp in *Tex19.1^−/−^* knockout testes. However by 19–22 dpp, a reduction in the number of meiotic and post-meiotic germ cells and an increase in the frequency of cell death are both evident in *Tex19.1^−/−^* knockout testes (Figures S3N, S4). In 22 dpp testes, clusters of TUNEL-positive cells can be seen within the layer of pachytene germ cells that line the lumen of the seminiferous tubule suggesting that at least some apoptosis is occurring at the pachytene stage of meiosis (Figure S3K–M). The high level of apoptosis in the *Tex19.1^−/−^* knockout testes increases by 29–31 dpp to the level seen in adult testes (Figure S3N). This data suggests that the reduction in the number of post-meiotic germ cells and increased levels of apoptosis seen in the adult *Tex19.1^−/−^* knockout testes is at least partly due to some *Tex19.1^−/−^* knockout germ cells initiating apoptosis during the pachytene stage of meiosis, and some *Tex19.1^−/−^* knockout germ cells initiating apoptosis during metaphase I.

Although the vast majority of the *Tex19.1^−/−^* null testes examined contained differentiating germ cells, two of thirty analysed knockout animals had an extremely severe phenotype with one testis that completely lacked germ cells. One of these agametic testes was isolated from a 31 dpp prepubertal mouse (Figure 4F) suggesting that this extreme phenotype is indicative of defects occuring during embryonic or early post-natal germ cell development rather than a progressive loss of spermatogonial stem cells in an ageing adult testis. However, as only a small number of testes exhibited this phenotype, we were not able to study this extreme phenotype further and instead focused on the meiotic phenotype evident in the vast majority of the *Tex19.1^−/−^* mutant testes.

### Chromosome Synapsis during Male Meiosis Is Impaired in the Absence of *Tex19.1*

**Figure 5:**
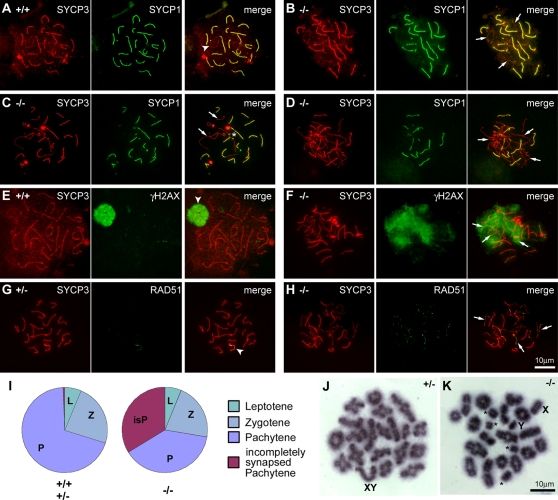
*Tex19.1^−/−^* null spermatocytes exhibit defects in chromosome synapsis during meiosis. (A–D) Synapsis of homologous chromosomes in meiotic cells; Sycp3 (red) is present on both synapsed and unsynapsed chromosomes during early meiotic prophase whereas Sycp1 (green) is only present on synapsed chromosomes. (A) In wild type pachytene cells all autosomal chromosomes are synapsed, and only the sex chromosomes remain unsynapsed (arrowhead). (B–D) Many *Tex19.1^−/−^* knockout cells exhibit incomplete synapsis where some chromosomes are unsynapsed (arrows) in cells that otherwise have pachytene appearance. Some unsynapsed chromosomes in *Tex19.1^−/−^* knockout cells, appear to form chains linked by regions of apparent non-homologous synapsis (asterisk) (E, F) Distribution of the DNA double-strand break marker γH2AX (green) in meiotic cells. (E) In wild type pachytene cells DNA double strand breaks that were generated during earlier stages of meiosis have been repaired leaving γH2AX staining restricted to the sex chromosomes (arrowhead). (F) In *Tex19.1^−/−^* knockout mice a proportion of pachytene-like cells exhibit incomplete resolution of γH2AX staining at places where incomplete synapsis is observed (arrows). (G, H) Distribution of Rad51 early recombination foci (green) in meiotic cells. (G) In normal pachytene cells, the early recombination marker Rad51 has already largely been lost from the fully synapsed autosomes but remains on the sex chromosomes (arrowhead). (H) In *Tex19.1^−/−^* knockout cells, Rad51 foci are abundant on unsynapsed chromosomes (arrows), but have mostly been lost from synapsed chromosomes (I) Distribution of meiotic stages in Sycp3/Sycp1-stained spreads. Around 100 chromosome spreads were scored from each of five *Tex19.1^−/−^* knockout animals and from each of five *Tex19.1^+/+^* wild-type or *Tex19.1^+/−^* heterozygous animals. Around half of the pachytene cells (47%) in *Tex19.1^−/−^* knockout testes feature incomplete synapsis. (J,K) Analysis of metaphase I chromosome spreads. (J) In normal metaphase I cells from *Tex19.1^+/−^* heterozygotes all pairs of homologous chromosomes are held together as bivalents by chiasmata. (K) Around two-thirds of the metaphase I spreads from *Tex19.1^−/−^* knockout mice exhibited univalent autosomal (asterisk) and/or sex (X,Y) chromosomes.

We next attempted to determine the cause of the increased apoptosis in *Tex19.1^−/−^* null testes. Defects in homologous chromosome synapsis or homologous recombination during meiotic prophase can cause apoptosis in late pachytene spermatocytes [31],[32]. Therefore we used immunocytochemistry on meiotic chromosome spreads to analyse chromosome synapsis and homologous recombination in *Tex19.1^−/−^* knockout testes. In order to analyse chromosome synapsis, meiotic chromosome spreads were stained using Sycp3 as a marker for lateral elements of meiotic chromosomes and Sycp1 as a marker for synapsed homologous chromosomes [6]. In wild-type pachytene cells the autosomal chromosome axes stain completely for both markers, whereas the X and Y sex chromosomes remain largely asynapsed with only a small area of Sycp1 staining in the pseudo-autosomal region (Figure 5A). In contrast, about half the pachytene cells in *Tex19.1^−/−^* homozygotes have Sycp3-stained autosomal chromosomal axes that lack Sycp1 staining (Figure 5B–D, I). The asynapsed chromosomes in *Tex19.1^−/−^* knockout cells did not appear to be arranged in homologous pairs (Figure 5D). However it is not clear whether the asynapsed chromosomes have never paired in *Tex19.1^−/−^* knockout spermatocytes, or have paired but have subsequently fallen apart. In some of the incompletely synapsed *Tex19.1^−/−^* knockout cells, some chromosomes appeared to form chains linked by regions of apparent non-homologous synapsis (Figure 5C, asterisk). Incompletely synapsed pachytene cells comprise less than 1% of spreads from *Tex19.1^+/+^* wild-type or *Tex19.1^+/−^* heterozygotous testes (Figure 5I). Thus *Tex19.1^−/−^* knockout animals exhibit defects in homologous chromosome synapsis during male meiosis.

During meiotic prophase, homologous recombination starts prior to homologous chromosome pairing and synapsis [33]. As progression of homologous recombination and chromosome synapsis are interdependent on each other [6],[7], we investigated whether the chromosome synapsis defect in *Tex19.1^−/−^* knockout spermatocytes was a consequence of an earlier defect in the initiation of homologous recombination. The appearance of DNA double strand breaks and the formation of early recombination foci during meiotic prophase can be detected by immunostaining for the phosphorylated histone γH2AX and the recombinase enzyme Rad51 respectively [33],[34]. γH2AX staining is normally present on chromatin during the leptotene and zygotene stages of early meiotic prophase. As synapsis proceeds during zygotene, the DNA double strand breaks are resolved, resulting in γH2AX staining disappearing from the autosomal chromosomes, but not the sex chromosomes. In normal *Tex19.1^+/+^* wild-type pachytene cells, chromosome synapsis is complete and only the sex chromosomes stain for γH2AX (Figure 5E). However, the incompletely synapsed pachytene cells in *Tex19.1^−/−^* knockout testes, exhibit strong diffuse γH2AX staining (Figure 5F). This γH2AX staining is localised to the regions of the chromosome spreads that contain the unsynapsed chromosomes (Figure 5F). Similarly, immunostaining for the early recombination foci marker Rad51, which largely disappears from autosomal chromosomes as synapsis proceeds, suggests that Rad51 foci are formed in *Tex19.1^−/−^* knockout spermatocytes, but are not resolved or matured on the unsynapsed chromosomes (Figure 5G, H). Thus the formation of DNA double strand breaks and the assembly of early recombination foci both appear to be occurring in *Tex19.1^−/−^* knockout spermatocytes. This suggests that the defect in meiotic chromosome synapsis that we have observed in *Tex19.1^−/−^* spermatocytes does not appear to be a secondary consequence of impaired initiation of homologous recombination. Rather, the presence of DNA double strand breaks and early recombination foci in the unsynapsed regions of the incompletely synapsed *Tex19.1^−/−^* pachytene spermatocytes is consistent with impaired chromosome synapsis. Furthermore, the presence of DNA double strand breaks and early recombination foci in unsynapsed regions of incompletely synapsed *Tex19.1^−/−^* pachytene spermatocytes indicates that the unsynapsed chromosomes arise from a failure to initiate synapsis rather than premature desynapsis. The unsynapsed chromosomes in the incompletely synapsed pachytene *Tex19.1^−/−^* knockout cells are presumably sufficient to trigger apoptosis at the pachytene checkpoint [31],[32], and would account for the increased levels of cell death seen in pachytene stage meiotic germ cells in *Tex19.1^−/−^* knockout testes (Figure S3).

Although incompletely synapsed pachytene cells could explain the increased levels of cell death in the pachytene meiotic germ cells in *Tex19.1^−/−^* knockout testes, the presence of apoptotic metaphase I spermatocytes in these animals suggests that there may be an additional defect later in spermatogenesis to account for cell death at the metaphase I stage. Around half of the *Tex19.1^−/−^* knockout pachytene cells did not appear to have any overt defects in chromosome synapsis (Figure 5I), and would therefore presumably be able to progress to metaphase I and continue through spermatogenesis. To investigate whether there might be additional defects in chromosome behaviour at later stages of meiosis in *Tex19.1^−/−^* knockout spermatocytes, we prepared and analysed meiotic metaphase I chromosome spreads. During metaphase I of meiosis, homologous chromosomes are held together as bivalents by chiasmata (Figure 5J). 94% of the metaphase I spreads from *Tex19.1^+/−^* heterozygous testes contained only bivalent metaphase I chromosomes, 5% contained univalent sex chromosomes, and 1% contained univalent autosomes. However, only 34% of the metaphase I spreads from *Tex19.1^−/−^* knockout testes contained only bivalent metaphase I chromosomes, while 56% of the spreads contained univalent sex chromosomes, and 33% contained univalent autosomes (Figure 5K). 23% of the *Tex19.1^−/−^* knockout metaphase I spreads feature univalent autosomes and univalent sex chromosomes. Thus *Tex19.1^−/−^* knockout testes contain increased numbers of univalent chromosomes at meiotic metaphase I that could potentially trigger apoptosis at the metaphase I checkpoint [35],[36] and account for the apoptotic metaphase I cells seen in *Tex19.1^−/−^* knockout testes. Furthermore, the presence of univalent chromosomes in metaphase I spreads from *Tex19.1^−/−^* knockout testes is indicative of a defect in the formation or maintenance of chiasmata in post-pachytene spermatocytes.

### Overexpression of Retrotransposable Elements in *Tex19.1^−/−^* Homozygous Testes

Meiotic defects similar to those present in the *Tex19.1^−/−^* knockout testes have been observed in various different mouse mutants that carry defects in genes encoding components of meiotic chromosomes, the meiotic recombination machinery, or the synaptonemal complex [6],[7]. However, we have been unable to detect any Tex19.1 protein physically associated with meiotic chromosomes by immunostaining (R.O., data not shown), and our finding that Tex19.1 is predominantly localised to the cytoplasm rather than the nucleus suggests that Tex19.1 is unlikely to be a component of meiotic chromosomes or the synaptonemal complex. We therefore reasoned that the meiotic defects present in the *Tex19.1^−/−^* knockout testes are unlikely to be a direct effect of Tex19.1 on meiotic chromosome structure or function but rather may be an indirect consequence of changes in meiotic gene expression.

In order to detect changes in gene expression in the testis of *Tex19.1^−/−^* knockout mice, we performed microarray analysis using an Illumina MouseWG-6 v1.1 Whole Genome Gene Expression Beadchip containing 48,318 different probes. To exclude potential differences in transcript levels due to the loss of post-meiotic germ cells in the *Tex19.1^−/−^* testes we performed this analysis on testes from 16 dpp prepubertal mice during the first synchronous wave of spermatogenesis. At this stage of testis development, some germ cells are already in the pachytene stage of meiosis, but no obvious changes in cell composition were apparent between *Tex19.1^+/+^* wild type and *Tex19.1^−/−^* knockout testes (Figure S4). RNAs from two different 16 dpp *Tex19.1^−/−^* knockout testes were compared with *Tex19.1^+/+^* wild type or *Tex19.1^+/−^* heterozygous littermates, and transcripts that had consistent and greater than three fold changes in relative gene expression between the two groups of animals were identified. The Mouse Genome Database (http://www.informatics.jax.org) currently lists 97 mutations that are known to give rise to meiotic arrest during spermatogenesis [8]. These male meiotic arrest genes include genes that encode components of meiotic chromosomes, the meiotic recombination machinery and the synaptonemal complex such as *Atm*, *Dmc1*, *γH2AX, Mlh1*, *Msh5*, *Rec8*, *Rad51*, *Smc1β*, *Spo11*, *Sycp1*, *Sycp2*, *Sycp3, Syce2* and *Tex14*. None of the male meiotic arrest genes listed in the Mouse Genome Database showed a consistent change in expression level in *Tex19.1^−/−^* knockout testes compared to littermate controls (I.R.A., data not shown). However, analysis of the microarray data suggested that the class II LTR-retrotransposon MMERVK10C [37] is upregulated by around four-fold in the testis RNA from each of the 16 dpp *Tex19.1^−/−^* knockout animals relative to their littermate controls (I.R.A., data not shown). The mouse genome contains around 16 approximately full-length copies of the MMERVK10C sequence in the genome, and a further 1200 fragments of the MMERVK10C endogenous retrovirus. Increased retrotransposon expression has been proposed to be responsible for impaired chromosome synapsis and meiotic defects during spermatogenesis in *Dnmt3L*, *Miwi2* and *Mili* mutant mice [11]–[14]. As overexpression of the MMERVK10C retrotransposons could similarly be responsible for the meiotic defects seen in *Tex19.1^−/−^* mutant mice we sought to determine whether MMERVK10C expression is indeed upregulated in the testis in the absence of *Tex19.1*.

**Figure 6:**
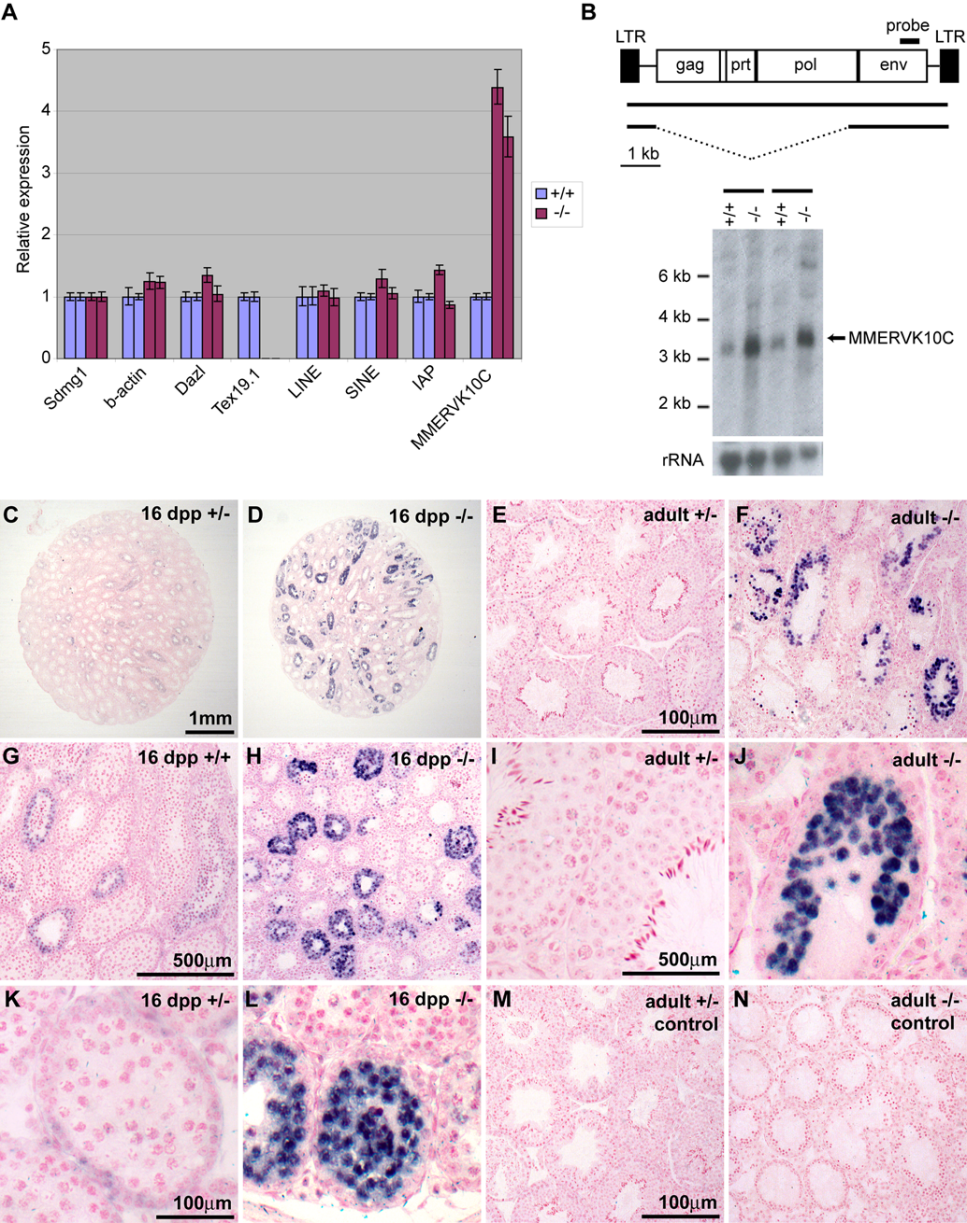
Expression of the endogenous retrovirus MMERVK10C is upregulated in *Tex19.1^−/−^* null testes. (A) Quantitative PCR showing relative expression of retrotransposons and marker genes in testes from 16 dpp *Tex19.1^−/−^* knockout animals relative to their *Tex19.1^+/+^* wild-type littermates. Expression levels in cDNAs prepared from different animals were normalised to *Sdmg1*, animals represented in the first and third columns were littermates, and those in the second and fourth columns were littermates. Error bars indicate standard error. (B) Northern blot probed for MMERVK10C *env* transcripts in *Tex19.1^+/+^* wild-type and *Tex19.1^−/−^* knockout testes at 16 dpp. A schematic diagram showing the organisation of the 8.5 kb full-length MMERVK10C element and the predicted size and organisation of the *env*-containing transcripts (∼8.5 kb and ∼3.3 kb, [39]) is shown above the Northern blot. Littermates are indicated with a black bar. 28S rRNA is shown as a loading control. (C–L) In situ hybridisation with an antisense MMERVK10C probe (purple precipitate) in *Tex19.1^+/+^*, *Tex19.1^+/−^* and *Tex19.1^−/−^* testes. Nuclei are counterstained with nuclear fast red. (C, D, G, H) Low-level expression of MMERVK10C can be seen in some seminiferous tubules in *Tex19.1^+/+^* and *Tex19.1^+/−^* testes. However, MMERVK10C transcripts are more abundant in testes from 16 dpp *Tex19.1^−/−^* animals than those from heterozygous or wild-type littermates. (K, L) MMERVK10C transcripts are upregulated in meiotic spermatocytes in 16 dpp *Tex19.1^−/−^* mutant testes. (E, F, I, J) MMERVK10C transcripts are upregulated in meiotic spermatocytes in testes from adult *Tex19.1* ^−/−^ knockout animals relative to their *Tex19.1^+/−^* heterozygous littermates. (M, N) Control in situ hybridisation with a sense MMERVK10C probe shows no staining in adult *Tex19.1^+/−^* or *Tex19.1^−/−^* testes.

The levels of MMERVK10C expression in testis cDNA from two *Tex19.1^−/−^* knockout animals relative to their *Tex19.1^+/+^* wild-type littermates were each tested by quantitative PCR (Figure 6A). The Sertoli cell marker *Sdmg1* [22] was used to normalise cDNAs from different animals. Although there was no significant change in the expression of the ubiquitously expressed β-actin gene, or the germ cell marker *Dazl* [38], expression of the MMERVK10C endogenous retrovirus was increased by a factor of approximately four-fold in both *Tex19.1^−/−^* knockout animals (Student's t-test, p<0.01) (Figure 6A). Expression of LINE, SINE or IAP retrotransposons showed no significant change in the absence of *Tex19.1* (Figure 6A).

To further validate the potential upregulation of MMERVK10C transcripts in the *Tex19.1^−/−^* knockout mice we performed Northern blots on testis RNA from the same two 16 dpp *Tex19.1^−/−^* knockout animals and their *Tex19.1^+/+^* wild type littermates. Using a probe derived from the *env* gene of the MMERVK10C endogenous retrovirus we were able to detect a predominant 3.2 kb MMERVK10C *env* transcript in mouse testes, and some weaker MMERVK10C *env* transcripts at around 4.5 kb and 7.5 kb (Figure 6B). The Northern blot profile for MMERVK10C *env* transcripts is comparable to that of *env*-containing transcripts from HERV-K endogenous retroviruses in human teratocarcinoma cell lines [39]. Northern blotting confirmed that the predominant 3.2 kb MMERVK10C *env* transcript is consistently more abundant in testes from *Tex19.1^−/−^* knockout animals than in testes from their wild-type littermates at 16 dpp (Figure 6B).

In order to determine which cell types are accumulating MMERVK10C transcripts in the *Tex19.1^−/−^* knockout testes we performed in situ hybridisation on testis sections using a MMERVK10C *env* probe (Figure 6C–N). In *Tex19.1^+/+^* wild-type and *Tex19.1^+/−^* heterozygous testes at 16 dpp, low levels of MMERVK10C *env* transcripts were present in some meiotic spermatocytes (Figure 6C,G). However, MMERVK10C transcripts were generally more abundant in *Tex19.1^−/−^* knockout testes than in testes from *Tex19.1^+/+^* wild-type or *Tex19.1^+/−^* heterozygous littermates at 16 dpp (Figure 6D, H). The increased levels of MMERVK10C *env* transcript in 16 dpp *Tex19.1^−/−^* testes appeared to be largely due to the presence of strongly expressing cells located towards the centre of the tubules where meiotic spermatocytes are present (Figure 6K, L). Similarly in adult animals MMERVK10C *env* transcripts were upregulated in meiotic germ cells in the testes from adult *Tex19.1^−/−^* knockout animals relative to their heterozygous littermates (Figure 6E, F, I, J). A total of nine different *Tex19.1^−/−^* knockout animals at various ages were assayed for MMERVK10C expression in the testes by in situ hybridisation, and MMERVK10C expression in *Tex19.1^−/−^* knockout testes was consistently higher that in *Tex19.1^+/+^* or *Tex19.1^+/−^* littermate controls. No in situ hybridisation signals were detected on testis sections using a sense MMERVK10C control probe (Figure 6M, N).

Taken together, the quantitative PCR, Northern blotting and in situ hybridisation data all suggest that transcripts from the MMERVK10C endogenous retrovirus are upregulated in the meiotic spermatocytes of *Tex19.1^−/−^* knockout testes.

The upregulation of retrotransposons in *Dnmt3L*, *Mili* and *Miwi2* mutant mice is associated with defects in de novo DNA methylation of IAP and LINE elements in the male germline, which presumably allows increased transcription of these elements during spermatogenesis [11]–[14]. In order to investigate whether the upregulation of MMERVK10C retrotransposons in *Tex19.1^−/−^* knockout testis was caused by a similar mechanism, we investigated the DNA methylation status of CpG dinucleotides in MMERVK10C elements by bisulphite sequencing MMERVK10C elements from 16 dpp prepubertal *Tex19.1^−/−^* knockout testes. The MMERVK10C element includes a weak CpG island overlapping the LTR and 5′untranslated region (Figure S5). As promoters with weak CpG islands are good candidates for regulation by DNA methylation [40], we examined DNA methylation at CpG dinucleotides within this region. Sequence analysis of 30 independent clones from each of *Tex19.1^+/+^* wild-type, *Tex19.1^+/−^* heterozygous and *Tex19.1^−/−^* homozygous 16 dpp testes showed that CpG dinucleotides in this region of the MMERVK10C element are predominantly methylated in the testis at this age (Figure S5). The MMERVK10C element was also methylated to a similarly high level in liver taken from the same animals as a somatic tissue control (Figure S5). The prepubertal testis is composed of approximately equal numbers of germ cells and somatic cells at 16 dpp [29],[30], therefore around half the clones analysed by bisulphite sequencing are likely to be derived from testicular germ cells and around half from testicular somatic cells. As all of the 16 dpp testis clones represented highly methylated DNA sequences (Figure S5), the MMERVK10C element appears to be highly methylated in both the germ cell and somatic cell compartments of *Tex19.1^+/+^* wild-type, *Tex19.1^+/−^* heterozygous and *Tex19.1^−/−^* homozygous 16 dpp testes.

Although we have been unable to find any evidence that the methylation status of MMERVK10C elements in the testis changes in the absence of *Tex19.1* (Figure S5), we cannot exclude the possibility that the absence of *Tex19.1* causes reduced DNA methylation in a subset of MMERVK10C elements in the genome, or in a subset of germ cells in 16 dpp testes. If only a subset of germ cells have altered DNA methylation at MMERVK10C elements in *Tex19.1^−/−^* mutant testes then we estimate that this subset would need to represent less than 25% of the germ cell population to be below our detection limit in this assay (χ^2^-test, p<0.05). Nevertheless, our observations that loss of *Tex19.1* causes the upregulation of MMERVK10C retrotransposon elements in the testis, but not IAP or LINE elements, combined with the absence of a detectable change in DNA methylation levels in MMERVK10C elements in *Tex19.1^−/−^* knockout testes, suggests that *Tex19.1*-mediated repression of retrotransposons may involve a mechanism that is distinct from *Dnmt3L*/*Miwi2*/*Mili*-mediated repression of retrotransposons. Thus we conclude that *Tex19.1* is part of a novel genetic pathway that represses retrotransposons in the male germline.

## Discussion

### The *Tex19.1^−/−^* Knockout Phenotype

This study describes the functional consequences of deleting the pluripotency-associated *Tex19.1* gene in mice. Our data shows that loss of *Tex19.1* causes impaired spermatogenesis and defects in chromosome synapsis during meiosis. Mutations in genes that are involved in various aspects of meiotic chromosome behaviour such as the initiation of recombination between homologous chromosomes, or the assembly of the synaptonemal complex, all typically cause defective chromosome synapsis during meiosis, and apoptosis in the male germline [6],[7]. However, although there is some similarity between these phenotypes and the *Tex19.1* mutant phenotype, we have been unable to detect any localisation of Tex19.1 to meiotic chromosomes by immunostaining testis sections or testis chromosome spreads (R.O., data not shown). Indeed our data suggest that Tex19.1 is a predominantly cytoplasmic protein and is therefore unlikely to play a direct role in meiotic chromosome behaviour. Thus, although *Tex19.1* mutant mice exhibit defects in chromosome pairing during meiosis, we do not believe that Tex19.1 is a component of meiotic chromosomes and favour the interpretation *Tex19.1* is influencing meiotic chromosome behaviour indirectly.

Our finding that Tex19.1 is a predominantly cytoplasmic protein in germ cells and embryonic stem cells contradicts a previous study suggesting that Tex19.1 is a nuclear protein in embryonic stem cells [17]. The reason for the discrepancy between these studies is not yet clear. Kuntz et al. [17] raised monoclonal antibodies to Tex19.1 and observed nuclear staining with those antibodies in embryonic stem cells and pre-implantation embryos. The Tex19.1 peptide used by Kuntz et al. [17] to raise the monoclonal anti-Tex19.1 antibody is located C-terminally to the peptide that we have used to raise the anti-Tex19.1 antibodies in this study. Both peptides, and indeed the entire *Tex19.1* open reading frame, lie within a single exon. In our study we have shown that Tex19.1 is predominantly cytoplasmic in embryonic stem cells by immunostaining and by Western blotting of subcellular fractions. We have also shown that Tex19.1 has a predominantly cytoplasmic localisation in germ cells by immunostaining germ cells isolated from embryonic testes, by immunohistochemistry on wax sections of adult testis and by Western blotting of subcellular fractions from prepubertal testes. Furthermore we have demonstrated the specificity of our antibody in the assays that we use by immunostaining and Western blotting on material from *Tex19.1^−/−^* knockout animals. As the cytoplasmic anti-Tex19.1 staining patterns that we present in this paper are lost in *Tex19.1^−/−^* knockout animals, at least some of the Tex19.1 protein that is present in germ cells and embryonic stem cells is cytoplasmic. However we cannot exclude the possibility that the two different antibodies raised in these two studies recognise mutually exclusive isoforms of Tex19.1 that have different subcellular localisations. Alternatively, the discrepancy between our study and the study by Kuntz et al. [17] could be caused by procedural differences, or by cross-reaction of anti-Tex19.1 antibodies with an unrelated antigen.

*Tex19.1^−/−^* null male mice showed some phenotypic variation between individuals ranging from completely agametic testes to fertility. This phenotypic variability may be partly due to the genetic heterogeneity in the outbred component of the genetic background used for this study. However, as some germ cells are more severely affected by the loss of *Tex19.1* than other germ cells in the same animal, there is also some phenotypic variability in the absence of genetic variation. Furthermore, our finding that loss of *Tex19.1* can impair spermatogenesis even in this heterogeneous genetic background suggests that mutations in the single human homologue, *TEX19*, could contribute to fertility problems in human populations. The human *TEX19* gene contains two premature stop codons in the open reading frame that truncates the Tex19 protein from 351 residues in mouse to 164 residues in human [17]. The first premature stop codon in the human *TEX19* gene is conserved in other primates suggesting that the C-terminal region of Tex19 is dispensable for function in primates [17]. The significance of this major difference in structure between human and mouse is at present unclear given our current level of understanding of the mechanisms underlying the phenotype in mouse.

The *Tex19* genomic locus has undergone a duplication event in rodents to generate two closely related divergently transcribed genes [17]. The mutation that we have engineered removes the entire *Tex19.1* open reading frame, but leaves *Tex19.2* intact. Therefore *Tex19.2* could potentially provide some functional redundancy with *Tex19.1*. Although *Tex19.1* and *Tex19.2* are reported to be expressed in testicular germ cells and testicular somatic cells respectively [17], there appears to be a moderate upregulation of *Tex19.2* in *Tex19.1^−/−^* knockout testes as judged by quantitative RT-PCR (I.R.A., data not shown). It is not clear at present whether this upregulation of *Tex19.2* occurs in the germ cells or somatic cells of the testis, but any upregulation of *Tex19.2* that is occurring does not seem to be able to fully compensate for loss of *Tex19.1*. Nevertheless, deletion of the entire *Tex19* locus may be required to rule out the possibility of some functional redundancy between these genes and may reveal additional functions for *Tex19.1* in the germline.

This study demonstrates that *Tex19.1* has a function in progression through meiosis in the male germline. Characterisation of the meiotic defect in *Tex19.1^−/−^* knockout spermatocytes indicates that homologous recombination is being initiated in the *Tex19.1^−/−^* knockout spermatocytes but that, for some chromosomes, synapsis does not occur. As homologous recombination and chromosome synapsis progress interdependently during meiosis, it is possible that the chromosome synapsis defect that we describe in *Tex19.1^−/−^* knockout spermatocytes is a secondary consequence of a defect in the progression of homologous recombination, or a secondary consequence of defects in the pairing between homologous chromosomes that normally precedes chromosome synapsis [7]. Further work is needed to dissect the molecular basis of the *Tex19.1* chromosome synapsis defect in more detail, and to understand if and how the upregulation of MMERVK10C retrotransposons that we detect in *Tex19.1^−/−^* spermatocytes causes these defects in meiotic chromosome synapsis.

### Tex19.1 and Repression of Retrotransposons in the Germline

The *Tex19.1* mutant phenotype bears some resemblance to the *Dnmt3L*, *Miwi2* and *Mili* mutant phenotypes in that they all exhibit defects in meiotic chromosome synapsis and increased expression of retrotransposons in the germline [11]–[14]. However it is not yet clear whether there is a direct causal relationship between these two events. The increase in retrotransposon expression does not appear to be caused by defects in meiotic chromosome synapsis [11],[13], but it is not clear whether or how the increase in retrotransposon expression causes the defects in meiotic chromosome synapsis in any of these mutant mice. Increased transposition of mobile genetic elements could introduce quantitative, qualitative, or temporal changes in the DNA double strand breaks normally present during early meiotic prophase that could interfere with the homologous recombination events that normally precede and initiate chromosome pairing. Support for this model comes from the observation that mutating genes involved in piRNA function in flies activates the DNA damage signalling pathway [41],[42]. Alternatively, it is possible that repression of retrotransposons is important for the fidelity of homolog pairing and synapsis during meiosis, and that increased expression of these repetitive elements either interferes with homolog recognition and synapsis, or promotes pairing between non-homologous chromosomes. A third possibility is that proteins encoded by the MMERVK10C endogenous retrovirus mediate the defects in meiotic chromosome synapsis by interfering with host cell proteins involved in meiotic chromosome behaviour or regulation of the meiotic cell cycle. In this regard it is important to note that transgenic mice expressing the rec protein derived from the HERVK human endogenous retrovirus exhibit defects in spermatogenesis [43]. Lastly, there may not be a direct causal relationship between retrotransposon de-repression and chromosome asynapsis. Rather the *Tex19.1*, *Dnmt3L*, *Miwi2* and *Mili* mutants may all cause defects in meiotic chromosome structure that lead to both retrotransposon de-repression and defective chromosome synapsis. Clearly further work is needed to clarify the molecular mechanism underlying the chromosome synapsis defect in the *Tex19.1* mutant mice presented here, and in the *Dnmt3L*, *Miwi2* and *Mili* mutant mice [11]–[13]. However, this study provides further evidence demonstrating a correlation between de-repression of retrotransposons and impaired chromosome synapsis during mouse meiosis.

Although there are gross similarities between the *Tex19.1* mutant phenotype and the *Dnmt3L*, *Miwi2* or *Mili* mutant phenotypes, there are also important differences. *Dnmt3L*, *Miwi2* and *Mili* are all required to repress LINE and IAP retrotransposons in the germline, and these three genes appear to converge on DNA methylation and transcriptional repression of these sequences in the genome [11]–[14]. However, repression of LINE and IAP retrotransposons is not perturbed in *Tex19.1^−/−^* knockout testes suggesting that *Tex19.1* is not involved in the transcriptional repression of LINE or IAP elements. Rather our data shows that transcripts from the MMERVK10C class of endogenous retroviruses accumulate in the germ cells in the absence of *Tex19.1*. These differences between the *Tex19.1* mutant phenotype and the *Dnmt3L*, *Miwi2* and *Mili* mutant phenotypes may reflect the existence of multiple mechanisms with different specificities to repress retrotransposons in the germline.

The *Tex19.1* mutant phenotype is characterised by the accumulation of MMERVK10C retrotransposon transcripts, but the molecular basis for this phenotype is not yet clear. The upregulation of MMERVK10C transcripts could be caused by changes acting at any level of gene expression from the initiation of transcription to mRNA turnover. We have not been able to find any difference in the level of DNA methylation at MMERVK10C elements in *Tex19.1* mutant testes. This provides further evidence that *Tex19.1* belongs to a different genetic pathway than *Miwi2*, *Mili* and *Dnmt3L* for repression of retrotransposons in the germline. However, we cannot exclude the possibility that DNA methylation may be altered in a subset of MMERVK10C elements in a subset of germ cells in the *Tex19.1* mutant testes, and that this subset of elements is responsible for the upregulation of MMERVK10C transcripts that we describe in the *Tex19.1* mutant testes. An alternative model to explain the upregulation of MMERVK10C elements in *Tex19.1* mutant testes is that Tex19.1 could be a transcriptional repressor of MMERVK10C elements. The nuclear localisation of Tex19.1 reported by Kuntz et al. [17] would be consistent with this type of mechanism operating. However, although we cannot exclude the possibility that some Tex19.1 acts in the nucleus in the germ cells in the adult testes, our finding that Tex19.1 is predominantly cytoplasmic in these cells would be more consistent with Tex19.1 acting to regulate gene expression at a post-transcriptional level. We are able to detect MMERVK10C transcripts in wild-type testes (Figure 6B,G) suggesting that some MMERVK10C transcripts must escape DNA methylation or transcriptional repression, and that post-transcriptional regulation of MMERVK10C mRNA may play a role in repressing the activity of this retrotransposon. The upregulation of MMERVK10C transcripts in *Tex19.1* mutant testes does not appear to be the result of changes in RNA splicing as the MMERVK10C isoforms present in *Tex19.1* mutant testes do not appear to be qualitatively different from those present in wild-type testes. However, the accumulation of MMERVK10C transcripts in *Tex19.1* knockout testes would be consistent with *Tex19.1* promoting degradation of MMERVK10C mRNA. Investigation into the biochemical function of Tex19.1 should provide a ready test of these models and generate some insight into the molecular mechanism of *Tex19.1-*dependent repression of MMERVK10C endogenous retroviruses.

Repression of retrotranposons in the mammalian germline requires mechanisms to distinguish retrotransposons from endogenous genes to allow repression to be targeted to the correct loci. piRNAs, a group of small RNAs that are physically associated with the piwi class of proteins, are abundant in male germ cells and some piRNAs have sequence homology to various classes of retrotransposon [14], [44]–[46]. The sequence homology between some piRNA molecules and retrotransposons is presumably used to target DNA methylation to retrotransposons rather than endogenous genes. Although there is good genetic evidence that the piwi class of proteins is involved in transcriptional repression of retrotransposons [12]–[14], there is also good biochemical evidence that piwi proteins and piRNAs are physically associated with the translational machinery in male germ cells [46],[47], suggesting a role in translation or mRNA turnover. Thus piRNA-mediated repression of retrotransposons may be working at multiple levels of gene expression in male germ cells. It will be informative to investigate whether the *Tex19.1* pathway for repression of retrotransposons that we describe here also utilises piRNAs to target repression to MMERVK10C elements.

One of the interesting aspects of the *Tex19.1* phenotype is that although the MMERVK10C subclass of retrotransposons is upregulated in *Tex19.1* mutant testes, LINE, SINE and IAP retrotransposons are not. It is not clear how *Tex19.1* determines specificity for the MMERVK10C element. Notably, IAP elements belong to the same subclass of endogenous retroviruses as MMERVK10C elements (class II LTR retrotransposons) but are not upregulated in *Tex19.1* mutant testes. Sequences within the MMERVK10C promoter or transcript could be involved in targeting *Tex19.1* activity to this element. Alternatively *Tex19.1* may have the potential to regulate a wider range of retrotransposons than we have been able to identify here, but alternative mechanisms to repress retrotransposon expression during spermatogenesis, such as DNA methylation, may limit the phenotypic effects of losing *Tex19.1* to a subset of its potential targets. Furthermore, as *Tex19.1* expression is not restricted to spermatogenesis but also occurs in primordial germ cells, oocytes and pluripotent stem cells, it will be of interest to determine if *Tex19.1* is involved in repressing MMERVK10C elements and other classes of retrotransposons in these cell types.

In addition to its role in the germline, *Tex19.1* is also expressed in pluripotent cells. Like germ cells, pluripotent cells are viable targets for retrotransposon activity as any new transposition events could be propagated through successive generations. Therefore pluripotent cells presumably also need to modulate retrotransposon activity to ensure that the mutational load on the genome is not too high. Our finding that *Tex19.1^−/−^* homozygotes are born at a sub-Mendelian frequency is consistent with a role for *Tex19.1* in pluripotent cells in early embryonic development. Further work is required to determine whether the loss of *Tex19.1^−/−^* homozygotes during embryogenesis is caused by defects in pluripotent cells, and whether pluripotent cells upregulate retrotransposon expression in *Tex19.1^−/−^* knockout embryos.

The ongoing battle between retrotransposons and the host genome has important consequences for evolution, and for genetic disease. Retrotransposons that can successfully evade genome defences in germ cells and pluripotent cells will be selected for during evolution, whereas germ cells and pluripotent cells are under selective pressure to keep the mutational load on the genome at sustainable levels. The striking differences in the relative abundance of different classes of retrotransposable elements between the mouse and human genomes suggest that this conflict is ongoing during mammalian evolution [9]. Although low levels of mutation and retrotransposition in the germline are required to generate the genetic variation essential for evolution, high levels of mutation or retrotransposition are deleterious to the survival of a species. In humans, endogenous retroviruses with intact coding sequences comprise a very small proportion of the genome [48], yet intact endogenous retroviral particles are found in human pluripotent stem cells, and in testicular germ cell tumours where the expression of endogenous retroviral proteins has been suggested to contribute to tumourigenesis [39],[43],[49]. Furthermore, a number of human genetic diseases are associated with de novo mutagenic retrotransposition events that disrupt the function of endogenous human genes [50],[51]. Our data suggests that *Tex19.1* is part of a mechanism that protects the genome from the deleterious effects of retrotransposon activity in the germline, and thereby helps to maintain genomic stability through successive generations.

## Supporting Information

Figure S1 Validation of anti-Tex19.1 antibody specificity. (A–C) In situ hybridisation for *Tex19.1* (purple precipitate) on adult testis sections. (A, C) In situ hybridisation with antisense *Tex19.1* probe gives signal in the outer region of seminiferous tubules where spermatogonia and early spermatocytes are localized. (B) Hybridisation with a sense probe does not lead to a signal. (D–F) Immunohistochemistry with anti-*Tex19.1* antibody (brown precipitate) on adult testis sections. (D) Anti-Tex19.1 antibodies stain spermatogonia and early spermatocytes in the outer region of the seminiferous tubules. (E) When the antibody is blocked with immunising peptide (+pep) the signal is not present. (F) Immunohistochemistry with anti-Tex19.1 antibodies on *Tex19.1^−/−^* knockout testes gives no signal. (G–I) Immunofluorescence on single cell suspensions from 14.5 dpc male embryonic gonads. Isolated gonads were trypsinised to single cell suspensions, then attached to poly lysine-coated slides, fixed with 4% paraformaldehyde in PBS and immunofluorescence was performed as described for cell spreads. Anti-Tex19.1 primary antibody is shown in green, nuclei counterstained with DAPI are shown in red. (G) Anti-Tex19.1 staining on a suspension of 14.5 dpc embryonic male gonadal cells gives strong signal in the germ cells. This signal is predominantly localized to the cytoplasm (inset in G). (H) This signal is not present when the antibody is blocked with immunising peptide (+pep). (I) Anti-Tex19.1 antibodies give no signal on gonadal cell suspensions from a 14.5 dpc male *Tex19.1^−/−^* knockout embryo.
(4.2 MB TIF)

Figure S2 Tex19.1 does not co-localise with the nuage marker Tdrd1 in the adult testis. Immunofluorescence staining of 6 µm thick wax sections of paraformaldehyde-fixed adult testis. (A–C) Anti-Tex19.1 antibodies (green) predominantly label the cytoplasm of spermatogonia (open arrowheads) and early spermatocytes (broad arrowheads). The anti-Tex19.1 antibodies are distributed throughout the cytoplasm of these cells. DNA is counterstained with DAPI (red). (D–F) Anti-Tdrd1 antibodies (green) label elaborate punctate cytoplasmic structures in spermatocytes (broad arrowheads) and a single cytoplasmic spot in round spermatids (narrow arrowheads). DNA is counterstained with DAPI (red).
(1.4 MB TIF)

Figure S3 *Tex19.1^−/−^* knockout animals exhibit increased levels of cell death in the testis. 6 µm thick wax sections of Bouin's-fixed testes were prepared, and the TUNEL assay for cell death performed using the DeadEnd Fluorometric TUNEL System (Promega) following the manufacturer's instructions. (A–M) TUNEL positive cells (green) in testes from *Tex19.1^−/−^* knockout animals and *Tex19.1^+/−^* heterozygous littermates. Nuclei are counterstained with DAPI (red). Panels G, J and M are merged images of panels E and F, and H and I, and K and L respectively. TUNEL-positive metaphase I cells (arrows) can be seen in some adult seminiferous tubules (E–G). Groups of TUNEL-positive cells (asterisks) can also be seen within the pachytene spermatocyte layer (arrowheads) of seminiferous tubules in adult (H–J) and prepubertal (K–M) testes. (N) *Tex19.1^−/−^* knockout testes have increased numbers of TUNEL-positive cells. For statistical analysis TUNEL-positive cells were counted in 25 seminiferous tubule cross-sections for each animal. At least three knockout and three wild-type or heterozygous animals were analysed at each age. Mean number of TUNEL-positive cells per 25 tubules and standard error are indicated. Mann Whitney U-test was used as a statistical test as the TUNEL positive cells are not normally distributed. *Tex19.1^−/−^* animals exhibit a statistically significant increase in the number of TUNEL-positive cells in the seminiferous tubules of the testis in 19–22 days post partum (dpp), 29–31 dpp, and in adult animals (Mann Whitney U-test, p<0.01) as indicated by asterisks.
(3.7 MB TIF)

Figure S4 Histology of *Tex19.1^−/−^* mutant testes during prepubertal development. Testis histology of *Tex19.1^−/−^* knockout pups during the first wave of spermatogenesis. (A, E) At 14 days post partum (dpp) some pachytene spermatocytes are present in both knockout and wild-type testes and no obvious difference can be seen between genotypes. (B, F) At 16 dpp more pachytene spermatocytes are present and there is no obvious difference between the cell types present in the testes of knockout and wild-type littermates. (C, G) By 20 dpp, the germ cells appear to be greatly reduced in number in *Tex19.1^−/−^* knockout testes (D, H) At 29 dpp, round spermatids and some elongating spermatids are present in heterozygous testes, but these cell types are reduced in number in testes from *Tex19.1^−/−^* knockout littermates.
(6.2 MB TIF)

Figure S5 MMERVK10C retrotransposons show no detectable change in DNA methylation status in *Tex19.1^−/−^* knockout testes. A schematic diagram showing the genomic organisation of the 5-end of the MMERVK10C retrotransposon is shown at the top of the figure. The long terminal repeat (LTR), 5′untranslated region (5′utr) and the start of the *gag* open reading frame are indicated, and the region analysed by bisulphite sequencing shown below with CpG dinucleotides indicated by grey circles. The DNA methylation status of CpG dinucleotides in 30 independent clones isolated from the liver or testes from 16 dpp *Tex19.1^+/+^* wild-type, *Tex19.1^+/−^* heterozygous and *Tex19.1^−/−^* homozygous animals is also shown. Black circles indicate methylated CpGs protected from bisulphite conversion, white circles indictate unmethylated CpGs. 500 ng genomic DNA from these tissues was bisulphite treated using the EZ DNA Methylation Gold kit (Zymo Research) then used as a template for nested PCR using the primers 5′-AGGTTTATAAAAGTAGTATTAG-3′ and 5′- ATAACAATTAAAACAATAACATA-3′, then 5′-TAAAAGTAGTATTAGTTTTGGG-3′ and 5′-AAACAAACAACACAATCCCA-3′. The resulting 480 bp PCR product was then blunt-end cloned into pBluescript II SK+ (Stratagene) and independent plasmid clones were isolated and sequenced. Around 95% of the non-CpG cytosine residues were converted to thymine in the analysed sequences indicating succesful bisulphite conversion.
(8.2 MB TIF)

Table S1 Primer sequences.
(0.02 MB DOC)

## References

[1] Lawson KA, Dunn NR, Roelen BAJ, Zeinstra LM, Davis AM (1999). Bmp4 is required for the generation of primordial germ cells in the mouse embryo.. Genes Dev.

[2] Lawson KA, Hage WJ (1994). Clonal analysis of the origin of primordial germ cells in the mouse.. Ciba Found Symp.

[3] Ying Y, Liu XM, Marble A, Lawson KA, Zhao GQ (2000). Requirement of Bmp8b for the Generation of Primordial Germ Cells in the Mouse.. Mol Endocrinol.

[4] Ohinata Y, Payer B, O'Carroll D, Ancelin K, Ono Y (2005). Blimp1 is a critical determinant of the germ cell lineage in mice.. Nature.

[5] Adams IR, McLaren A (2002). Sexually dimorphic development of mouse primordial germ cells: switching from oogenesis to spermatogenesis.. Development.

[6] Costa Y, Cooke H (2007). Dissecting the mammalian synaptonemal complex using targeted mutations.. Chromosome Res.

[7] Page SL, Hawley RS (2004). The Genetics and Molecular Biology of the Synaptonemal Complex.. Annu Rev Cell Dev Biol.

[8] Bult CJ, Eppig JT, Kadin JA, Richardson JE, Blake JA, the Mouse Genome Database Group (2008). The Mouse Genome Database (MGD): mouse biology and model systems.. Nucleic Acids Res.

[9] Mouse Genome Sequencing Consortium (2002). Initial sequencing and comparative analysis of the mouse genome.. Nature.

[10] De La Fuente R, Baumann C, Fan T, Schmidtmann A, Dobrinski I (2006). Lsh is required for meiotic chromosome synapsis and retrotransposon silencing in female germ cells.. Nat Cell Biol.

[11] Bourc'his D, Bestor TH (2004). Meiotic catastrophe and retrotransposon reactivation in male germ cells lacking Dnmt3L.. Nature.

[12] Aravin AA, Sachidanandam R, Girard A, Fejes-Toth K, Hannon GJ (2007). Developmentally Regulated piRNA Clusters Implicate MILI in Transposon Control.. Science.

[13] Carmell MA, Girard A, van de Kant HJG, Bourc'his D, Bestor TH (2007). MIWI2 Is Essential for Spermatogenesis and Repression of Transposons in the Mouse Male Germline.. Dev Cell.

[14] Kuramochi-Miyagawa S, Watanabe T, Gotoh K, Totoki Y, Toyoda A (2008). DNA methylation of retrotransposon genes is regulated by Piwi family members MILI and MIWI2 in murine fetal testes.. Genes Dev.

[15] Wang PJ, McCarrey JR, Yang F, Page DC (2001). An abundance of X-linked genes expressed in spermatogonia.. Nat Genet.

[16] Reynolds N, Collier B, Maratou K, Bingham V (2005). Dazl binds in vivo to specific transcripts and can regulate the pre-meiotic translation of Mvh in germ cells.. Hum Mol Genet.

[17] Kuntz S, Kieffer E, Bianchetti L, Lamoureux N, Fuhrmann G (2008). Tex19, a Mammalian-Specific Protein with a Restricted Expression in Pluripotent Stem Cells and Germ Line.. Stem Cells.

[18] D'Amour KA, Gage FH (2003). Genetic and functional differences between multipotent neural and pluripotent embryonic stem cells.. Proc Natl Acad Sci U S A.

[19] Joyner AL (2000). Gene Targeting: A Practical Approach..

[20] Harlow E, Lane D (1988). Antibodies : a laboratory manual..

[21] Livak KJ, Schmittgen TD (2001). Analysis of Relative Gene Expression Data Using Real-Time Quantitative PCR and the 2^−ΔΔCT^ Method.. Methods.

[22] Best D, Sahlender DA, Walther N, Peden AA, Adams IR (2008). Sdmg1 is a conserved transmembrane protein associated with germ cell sex determination and germline-soma interactions in mice.. Development.

[23] Costa Y, Speed R, Ollinger R, Alsheimer M, Semple CA (2005). Two novel proteins recruited by synaptonemal complex protein 1 (SYCP1) are at the centre of meiosis.. J Cell Sci.

[24] Chandley AC, Speed RM, Ma K, Gosden JR (1994). Meiotic chromosome preparation.. Chromosome Analysis Protocols.

[25] Meehan T, Schlatt S, O'Bryan MK, de Kretser DM, Loveland KL (2000). Regulation of germ cell and Sertoli cell development by activin, follistatin and FSH.. Dev Biol.

[26] Russell LD, Ettlin RA, SinhaHikim AP, Clegg ED (1990). Histological and Histopathological Evaluation of the Testis..

[27] Wang PJ, Page DC, McCarrey JR (2005). Differential expression of sex-linked and autosomal germ-cell-specific genes during spermatogenesis in the mouse.. Hum Mol Genet.

[28] Chuma S, Hosokawa M, Kitamura K, Kasai S, Fujioka M (2006). Tdrd1/Mtr-1, a tudor-related gene, is essential for male germ-cell differentiation and nuage/germinal granule formation in mice.. Proc Natl Acad Sci U S A.

[29] Bellve AR, Cavicchia JC, Millette CF, O'Brien DA, Bhatnagar YM (1977). Spermatogenic cells of the prepuberal mouse. Isolation and morphological characterization.. J Cell Biol.

[30] Ellis PJI, Furlong RA, Wilson A, Morris S, Carter D (2004). Modulation of the mouse testis transcriptome during postnatal development and in selected models of male infertility.. Mol Hum Reprod.

[31] de Rooij DG, de Boer P (2003). Specific arrests of spermatogenesis in genetically modified and mutant mice.. Cytogenet Genome Res.

[32] Hamer G, Novak I, Kouznetsova A, Hoog C (2008). Disruption of pairing and synapsis of chromosomes causes stage-specific apoptosis of male meiotic cells.. Theriogenology.

[33] Mahadevaiah SK, Turner JMA, Baudat F, Rogakou EP, de Boer P (2001). Recombinational DNA double-strand breaks in mice precede synapsis.. Nat Genet.

[34] Barlow AL, Benson FE, West SC, Hulten MA (1997). Distribution of the Rad51 recombinase in human and mouse spermatocytes.. EMBO J.

[35] Odorisio T, Rodriguez TA, Evans EP, Clarke AR (1998). The meiotic checkpoint monitoring sypapsis eliminates spermatocytes via p53-independent apoptosis.. Nat Genet.

[36] Eaker S, Cobb J, Pyle A, Handel MA (2002). Meiotic Prophase Abnormalities and Metaphase Cell Death in MLH1-Deficient Mouse Spermatocytes: Insights into Regulation of Spermatogenic Progress.. Dev Biol.

[37] Jurka J, Kapitononv VV, Klonowski P, Kohany O (2005). Repbase Update, a database of eukaryotic repetitive elements.. Cytogenet Genome Res.

[38] Ruggiu M, Speed R, Taggart M, McKay SJ, Kilanowski F (1997). The mouse *Dazla* gene encodes a cytoplasmic protein essential for gametogenesis.. Nature.

[39] Lower R, Boller K, Hasenmaier B, Korbmacher C, Muller-Lantzsch N (1993). Identification of Human Endogenous Retroviruses with Complex mRNA Expression and Particle Formation.. Proc Natl Acad Sci U S A.

[40] Weber M, Hellmann I, Stadler MB, Ramos L, Paabo S (2007). Distribution, silencing potential and evolutionary impact of promoter DNA methylation in the human genome.. Nat Genet.

[41] Chen Y, Pane A, Schupbach T (2007). cutoff and aubergine Mutations Result in Retrotransposon Upregulation and Checkpoint Activation in Drosophila.. Curr Biol.

[42] Klattenhoff C, Bratu DP, McGinnis-Schultz N, Koppetsch BS, Cook HA (2007). Drosophila rasiRNA Pathway Mutations Disrupt Embryonic Axis Specification through Activation of an ATR/Chk2 DNA Damage Response.. Dev Cell.

[43] Galli UM, Sauter M, Lecher B, Maurer S, Herbst H (2005). Human endogenous retrovirus rec interferes with germ cell development in mice and may cause carcinoma in situ, the predecessor lesion of germ cell tumors.. Oncogene.

[44] Aravin A, Gaidatzis D, Pfeffer S, Lagos-Quintana M, Landgraf P (2006). A novel class of small RNAs bind to MILI protein in mouse testes.. Nature.

[45] Girard A, Sachidanandam R, Hannon GJ, Carmell MA (2006). A germline-specific class of small RNAs binds mammalian Piwi proteins.. Nature.

[46] Grivna ST, Beyret E, Wang Z, Lin H (2006). A novel class of small RNAs in mouse spermatogenic cells.. Genes Dev.

[47] Grivna ST, Pyhtila B, Lin H (2006). MIWI associates with translational machinery and PIWI-interacting RNAs (piRNAs) in regulating spermatogenesis.. Proc Natl Acad Sci U S A.

[48] International Human Genome Sequencing Consortium (2001). Initial sequencing and analysis of the human genome.. Nature.

[49] Bronson DL, Saxinger WC, Ritzi DM, Fraley EE (1984). Production of Virions with Retrovirus Morphology by Human Embryonal Carcinoma Cells in vitro.. J Gen Virol.

[50] Maksakova IA, Romanish MT, Gagnier L, Dunn CA, van de Lagemaat LN (2006). Retroviral Elements and Their Hosts: Insertional Mutagenesis in the Mouse Germ Line.. PLoS Genet.

[51] Chen JM, Stenson P, Cooper D, Férec C (2005). A systematic analysis of LINE-1 endonuclease-dependent retrotranspositional events causing human genetic disease.. Hum Genet.

