# Supplementary figures and images for: Deletion of the Pluripotency-Associated Tex19.1 Gene Causes Activation of Endogenous Retroviruses and Defective Spermatogenesis in Mice
Source: PLoS Genet. 2008 Sep 19;4(9):e1000199. doi: 10.1371/journal.pgen.1000199 (PMC2531233; doi:10.1371/journal.pgen.1000199)

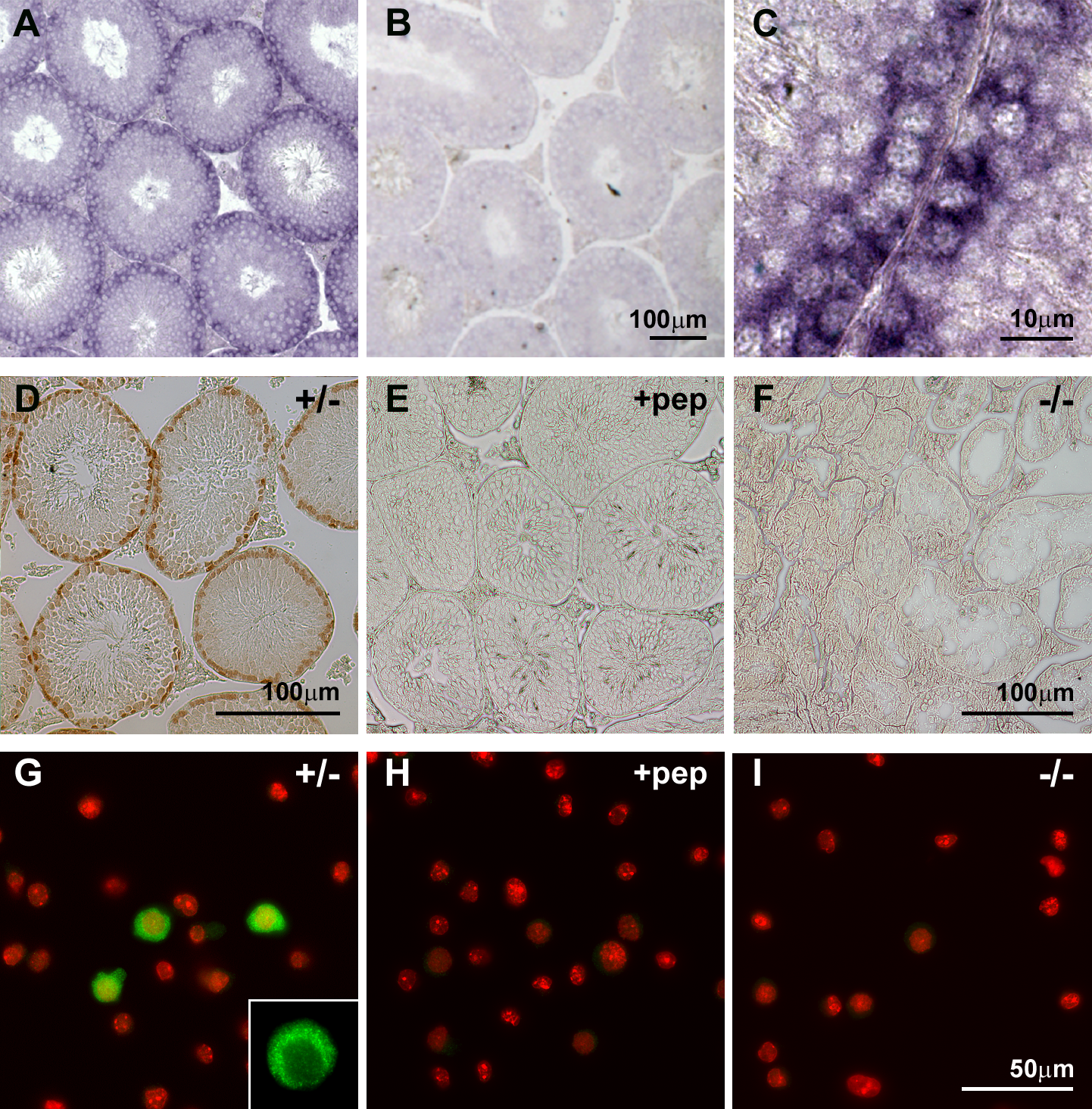

Supplement: Figure S1 — Validation of anti-Tex19.1 antibody specificity. (A–C) In situ hybridisation for Tex19.1 (purple precipitate) on adult testis sections. (A, C) In situ hybridisation with antisense Tex19.1 probe gives signal in the outer region of seminiferous tubules where spermatogonia and early spermatocytes are localized. (B) Hybridisation with a sense probe does not lead to a signal. (D–F) Immunohistochemistry with anti-Tex19.1 antibody (brown precipitate) on adult testis sections. (D) Anti-Tex19.1 antibodies stain spermatogonia and early spermatocytes in the outer region of the seminiferous tubules. (E) When the antibody is blocked with immunising peptide (+pep) the signal is not present. (F) Immunohistochemistry with anti-Tex19.1 antibodies on Tex19.1−/− knockout testes gives no signal. (G–I) Immunofluorescence on single cell suspensions from 14.5 dpc male embryonic gonads. Isolated gonads were trypsinised to single cell suspensions, then attached to poly lysine-coated slides, fixed with 4% paraformaldehyde in PBS and immunofluorescence was performed as described for cell spreads. Anti-Tex19.1 primary antibody is shown in green, nuclei counterstained with DAPI are shown in red. (G) Anti-Tex19.1 staining on a suspension of 14.5 dpc embryonic male gonadal cells gives strong signal in the germ cells. This signal is predominantly localized to the cytoplasm (inset in G). (H) This signal is not present when the antibody is blocked with immunising peptide (+pep). (I) Anti-Tex19.1 antibodies give no signal on gonadal cell suspensions from a 14.5 dpc male Tex19.1−/− knockout embryo. (4.2 MB TIF) [file pgen.1000199.s001.tif]

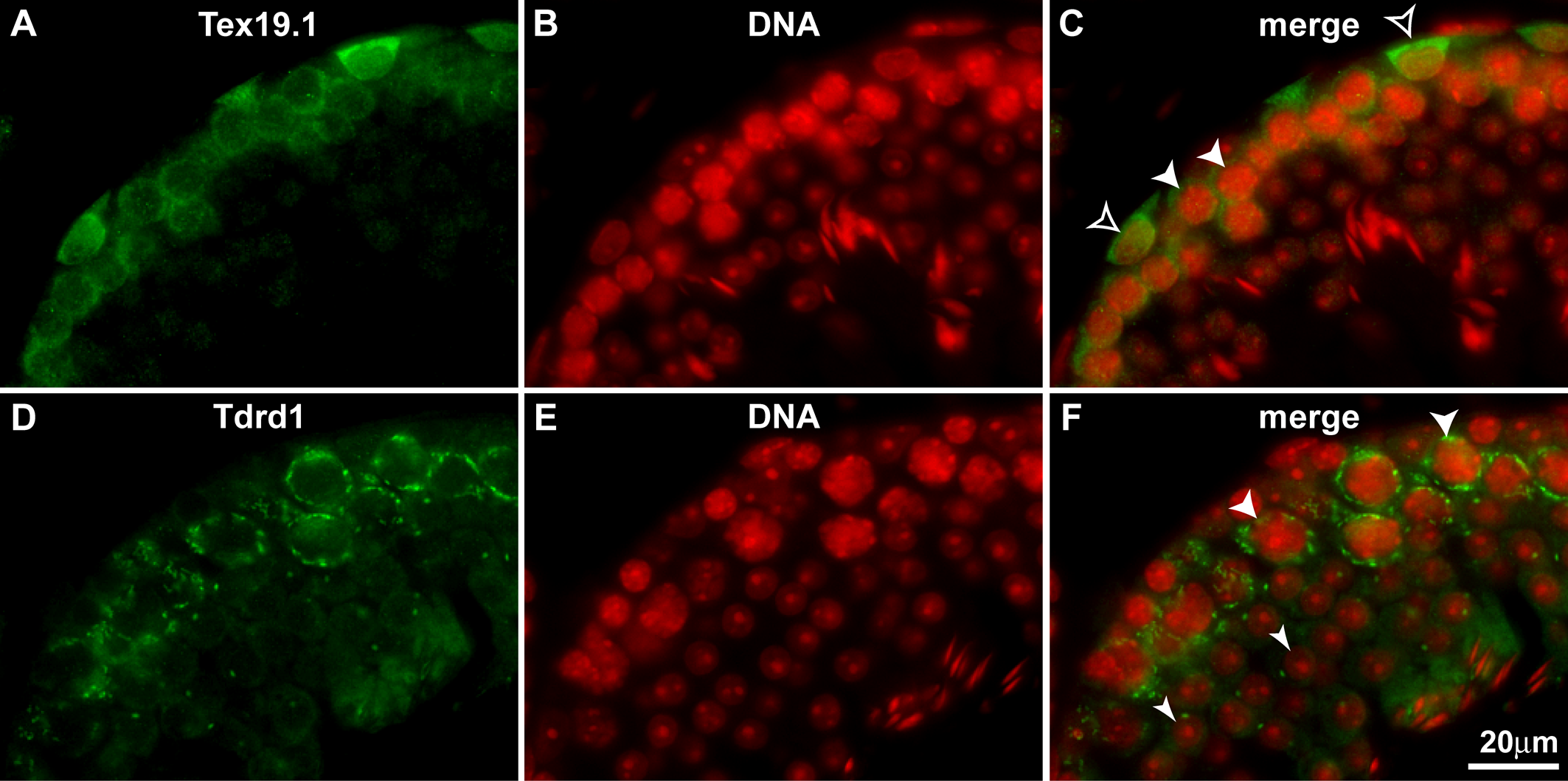

Supplement: Figure S2 — Tex19.1 does not co-localise with the nuage marker Tdrd1 in the adult testis. Immunofluorescence staining of 6 µm thick wax sections of paraformaldehyde-fixed adult testis. (A–C) Anti-Tex19.1 antibodies (green) predominantly label the cytoplasm of spermatogonia (open arrowheads) and early spermatocytes (broad arrowheads). The anti-Tex19.1 antibodies are distributed throughout the cytoplasm of these cells. DNA is counterstained with DAPI (red). (D–F) Anti-Tdrd1 antibodies (green) label elaborate punctate cytoplasmic structures in spermatocytes (broad arrowheads) and a single cytoplasmic spot in round spermatids (narrow arrowheads). DNA is counterstained with DAPI (red). (1.4 MB TIF) [file pgen.1000199.s002.tif]

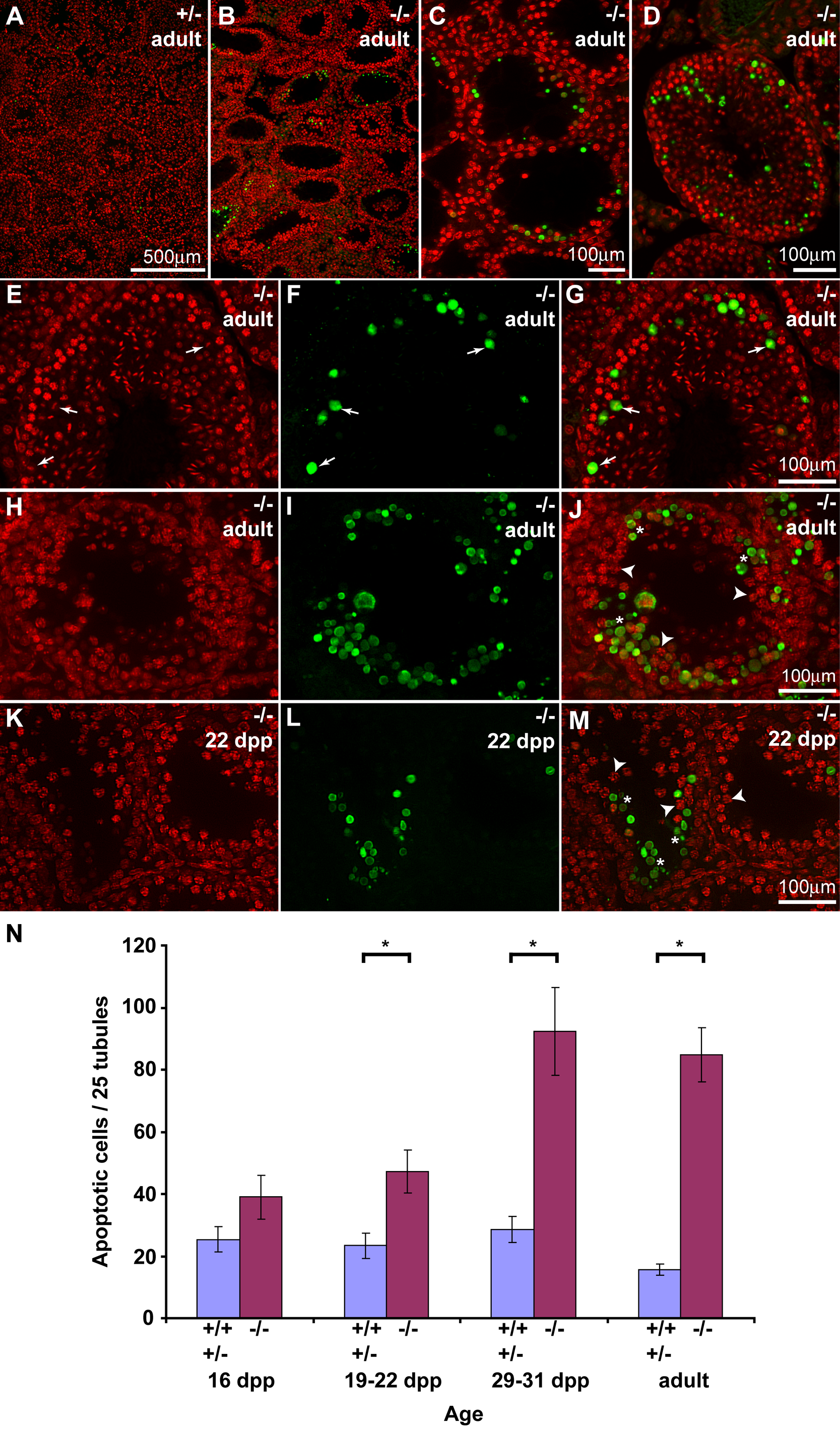

Supplement: Figure S3 — Tex19.1−/− knockout animals exhibit increased levels of cell death in the testis. 6 µm thick wax sections of Bouin's-fixed testes were prepared, and the TUNEL assay for cell death performed using the DeadEnd Fluorometric TUNEL System (Promega) following the manufacturer's instructions. (A–M) TUNEL positive cells (green) in testes from Tex19.1−/− knockout animals and Tex19.1+/− heterozygous littermates. Nuclei are counterstained with DAPI (red). Panels G, J and M are merged images of panels E and F, and H and I, and K and L respectively. TUNEL-positive metaphase I cells (arrows) can be seen in some adult seminiferous tubules (E–G). Groups of TUNEL-positive cells (asterisks) can also be seen within the pachytene spermatocyte layer (arrowheads) of seminiferous tubules in adult (H–J) and prepubertal (K–M) testes. (N) Tex19.1−/− knockout testes have increased numbers of TUNEL-positive cells. For statistical analysis TUNEL-positive cells were counted in 25 seminiferous tubule cross-sections for each animal. At least three knockout and three wild-type or heterozygous animals were analysed at each age. Mean number of TUNEL-positive cells per 25 tubules and standard error are indicated. Mann Whitney U-test was used as a statistical test as the TUNEL positive cells are not normally distributed. Tex19.1−/− animals exhibit a statistically significant increase in the number of TUNEL-positive cells in the seminiferous tubules of the testis in 19–22 days post partum (dpp), 29–31 dpp, and in adult animals (Mann Whitney U-test, p<0.01) as indicated by asterisks. (3.7 MB TIF) [file pgen.1000199.s003.tif]

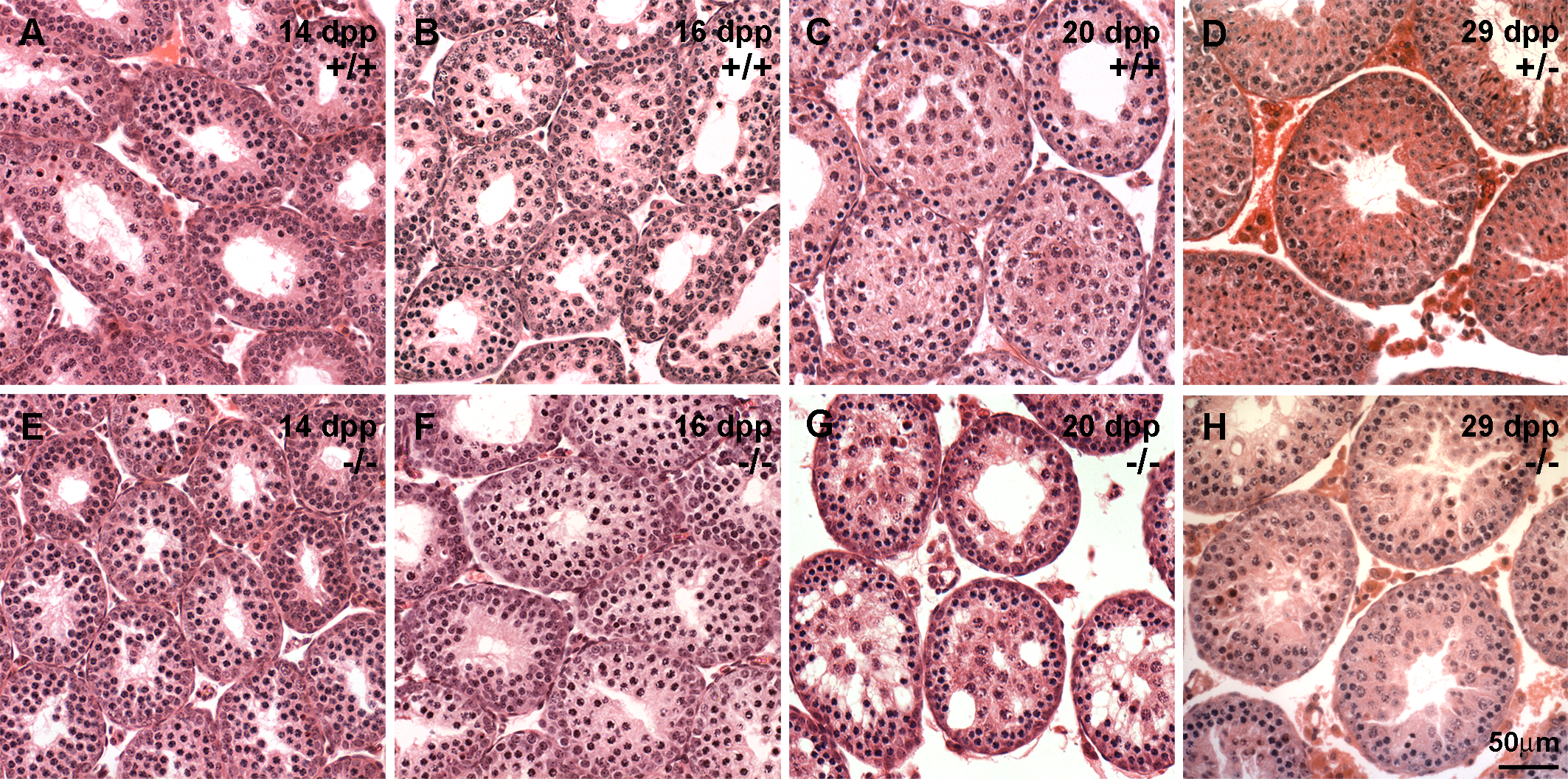

Supplement: Figure S4 — Histology of Tex19.1−/− mutant testes during prepubertal development. Testis histology of Tex19.1−/− knockout pups during the first wave of spermatogenesis. (A, E) At 14 days post partum (dpp) some pachytene spermatocytes are present in both knockout and wild-type testes and no obvious difference can be seen between genotypes. (B, F) At 16 dpp more pachytene spermatocytes are present and there is no obvious difference between the cell types present in the testes of knockout and wild-type littermates. (C, G) By 20 dpp, the germ cells appear to be greatly reduced in number in Tex19.1−/− knockout testes (D, H) At 29 dpp, round spermatids and some elongating spermatids are present in heterozygous testes, but these cell types are reduced in number in testes from Tex19.1−/− knockout littermates. (6.2 MB TIF) [file pgen.1000199.s004.tif]

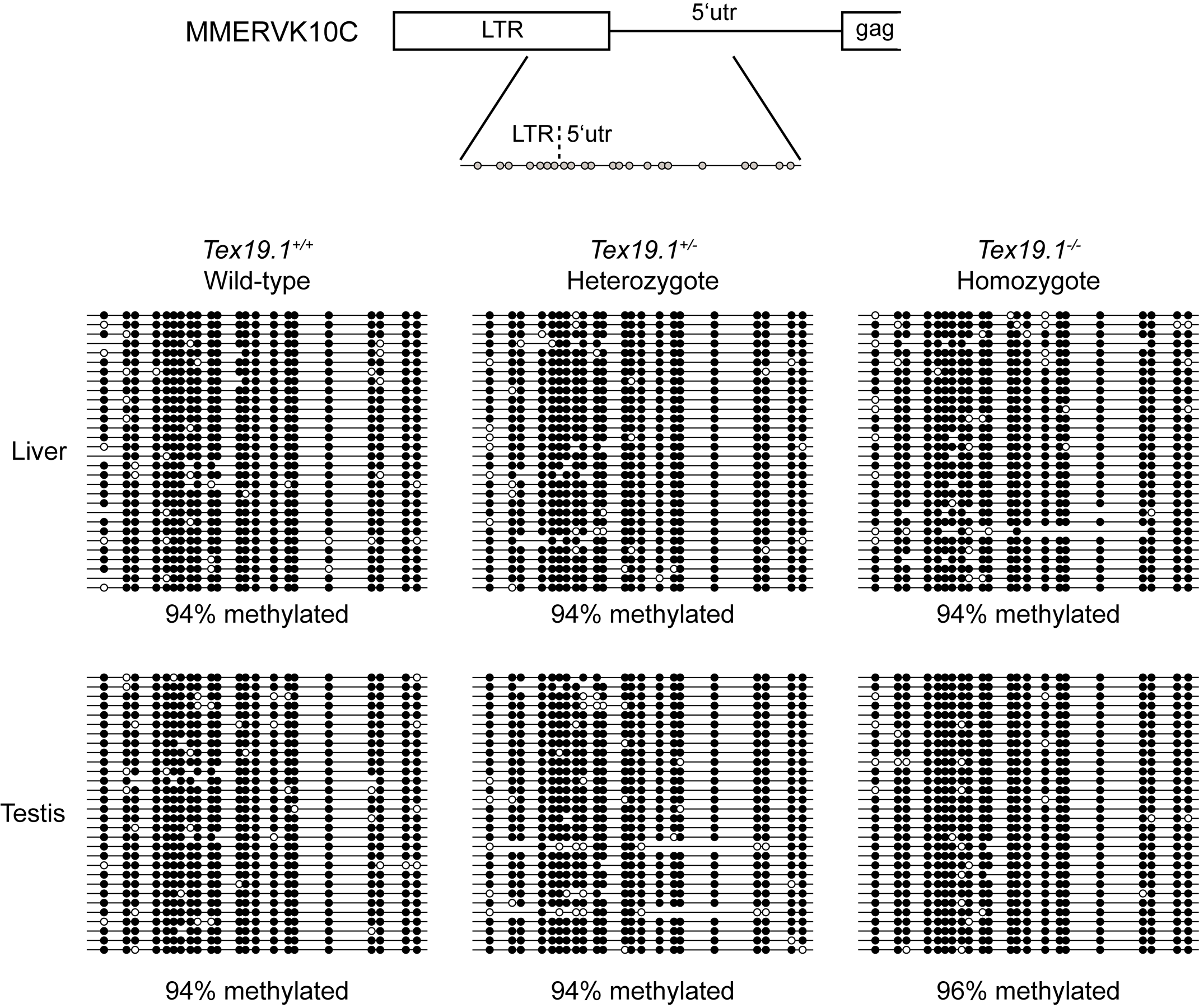

Supplement: Figure S5 — MMERVK10C retrotransposons show no detectable change in DNA methylation status in Tex19.1−/− knockout testes. A schematic diagram showing the genomic organisation of the 5-end of the MMERVK10C retrotransposon is shown at the top of the figure. The long terminal repeat (LTR), 5′untranslated region (5′utr) and the start of the gag open reading frame are indicated, and the region analysed by bisulphite sequencing shown below with CpG dinucleotides indicated by grey circles. The DNA methylation status of CpG dinucleotides in 30 independent clones isolated from the liver or testes from 16 dpp Tex19.1+/+ wild-type, Tex19.1+/− heterozygous and Tex19.1−/− homozygous animals is also shown. Black circles indicate methylated CpGs protected from bisulphite conversion, white circles indictate unmethylated CpGs. 500 ng genomic DNA from these tissues was bisulphite treated using the EZ DNA Methylation Gold kit (Zymo Research) then used as a template for nested PCR using the primers 5′-AGGTTTATAAAAGTAGTATTAG-3′ and 5′- ATAACAATTAAAACAATAACATA-3′, then 5′-TAAAAGTAGTATTAGTTTTGGG-3′ and 5′-AAACAAACAACACAATCCCA-3′. The resulting 480 bp PCR product was then blunt-end cloned into pBluescript II SK+ (Stratagene) and independent plasmid clones were isolated and sequenced. Around 95% of the non-CpG cytosine residues were converted to thymine in the analysed sequences indicating succesful bisulphite conversion. (8.2 MB TIF) [file pgen.1000199.s005.tif]
