# Supplementary material for: Deletion of the Pluripotency-Associated Tex19.1 Gene Causes Activation of Endogenous Retroviruses and Defective Spermatogenesis in Mice
Source: PLoS Genet. 2008 Sep 19;4(9):e1000199. doi: 10.1371/journal.pgen.1000199 (PMC2531233; doi:10.1371/journal.pgen.1000199)
Supplement: Table S1 — Primer sequences. (0.02 MB DOC) [file pgen.1000199.s006.doc]

| **Gene** | **Purpose of primer** | Primer Sequences |
| --- | --- | --- |
| *Tex19.1* | To clone short homology arm | 5’-TAGCCTCACTCCCCAAGATG-3’  5’-CGATGCCCTATTCTGGTGTC-3’ |
| *Tex19.1* | To clone long homology arm | 5’-GTGCTCCTTTTGAGGGCTTT-3’  5’-AGGTTGGCTAATGTCGCGTA-3’ |
| *Tex19.1* | To genotype *Tex19.1* wild-type allele | 5’-CTTCAGGAGGTCTGATGCCCTCT-3’  5’-GAGTGTTGTGTGGTGGGTGTTATGG-3’ |
| *Tex19.1* | To genotype *Tex19.1* knockout allele | 5’-CACCGCCTGTGCTCTAGTAGCTT-3’  5’-CTTCAGGAGGTCTGATGCCCTCT-3’ |
| *Tex19.1* | To generate probe for Southern blot | 5’-TCTGTCATAAGGGTGGCTTG-3’  5’-GGCTGGGTCATATCCAGTG-3’ |
| *Tex19.1* | RT-PCR | *5’-* GCCGGTACTGTAGAGGCAAA-3’  5’-CTCGATGGACTGAGGAGGTC-3’ |
| *Gapdh* | RT-PCR | 5’-CCTGCGACTTCAACAGCAACTCCCA-3’  5’-TGAGGTCCACCACCCTGTTGCTGTA-3’ |
| MMERVK10C | To generate probe for Northern blot | 5’-CCTGCGACTTCAACAGCAACTCCCA-3’  5’-TGAGGTCCACCACCCTGTTGCTGTA-3’ |
| *Sdmg1* | qPCR | 5’-AGTGGCTTAAAGGAGACCATCA-3’  5’- TCAGCATGCGTTTCTCTATGTT-3’ |
| β-actin | qPCR | 5’-GGCTGTATTCCCCTCCATCG-3’  5’-ACATGGCATTGTTACCAACTGG-3’ |
| Dazl | qPCR | 5’-tctttgccagatatggctcagt-3’  5’-cttctgcacatccacgtcatta-3’ |
| Tex19.1 | qPCR | 5’-aaaatgggccacccacatctc-3’  5’-ccactggcccttggaccagac-3’ |
| LINE1 ORF2 | qPCR | 5’-ggagggacatttcattctcatc-3’  5’-gctgctcttgtatttggagcataga-3’ |
| SINE B1 | qPCR | 5’-tggtggtgcatgcctttaat-3’  5’-cctggtgtcctggaactcact-3’ |
| IAP-B | qPCR | 5’-gcaccctcaaagcctatcttat-3’  5’-tcccttggtcagtctggattt-3’ |
| MMERVK10C | qPCR | 5’-GGTAAAGTCTCCGAGGGTCA-3’  5’-AACTGGTCGCAGGAGCTG-3’ |

**Supplementary Table S1. Primer sequences**
